# Semisynthetic Derivatives of Polygodial as α-Glucosidase and α-Amylase Inhibitors: In Vitro Evaluation, Molecular Docking and Molecular Dynamics Simulation

**DOI:** 10.3390/pharmaceutics18080960

**Published:** 2026-08-05

**Authors:** Viviana Burgos, Cecilia Villegas, Carlos Sanzana, Benjamin Oporto, Bernd Schmidt, Vaderament-Alexe Nchiozem-Ngnitedem, Muhammad Javid Iqbal, Cristian Paz

**Affiliations:** 1 Escuela de Tecnología Médica, Facultad de Salud, Universidad Santo Tomás, Temuco 4780000, Chile; vburgos7@santotomas.cl; 2 Departamento de Ciencias Biológicas y Químicas, Facultad de Recursos Naturales, Universidad Católica de Temuco, Rudecindo Ortega, Temuco 4780000, Chile; cecilia.villegas@uct.cl; 3 Laboratory of Natural Products & Drug Discovery, Department of Basic Sciences, Faculty of Medicine, Universidad de La Frontera, Temuco 4780000, Chile; c.sanzana02@ufromail.cl (C.S.); b.oporto02@ufromail.cl (B.O.); 4 Institut für Chemie, Universität Potsdam, Karl-Liebknecht-Str. 24-25, D-14476 Potsdam, Germany; bernd.schmidt@uni-potsdam.de (B.S.); n.vaderamentalexe@gmail.com (V.-A.N.-N.)

**Keywords:** polygodial, drimane sesquiterpenoids, α-glucosidase inhibition, α-amylase inhibition, molecular docking, molecular dynamics simulation, *Drimys winteri*

## Abstract

**Background**: Polygodial (**9**), a drimane sesquiterpene dialdehyde from *Drimys winteri*, has not previously been examined against carbohydrate-hydrolyzing enzymes. **Methods**: Regioselective Wittig olefination at C12 gave the enoate **10**; reduction of the remaining C11 aldehyde with NaBH_4_ was followed by spontaneous intramolecular conjugate addition, affording the annellated tetrahydrofuran **12a** and the bridged ether **12b**. **Results**: All three derivatives inhibited α-glucosidase and α-amylase more strongly than the parent compound. Compound **12a** was the most active α-glucosidase inhibitor (IC_50_ = 53.98 ± 3.0 µM, against 90.36 ± 4.0 µM for acarbose) and, in docking, the only derivative to occupy the acarbose-binding site of the enzyme (−8.1 kcal/mol). However, this **12a**–α-glucosidase pose was not maintained during the 200 ns simulations. The enoate **10** was the most active α-amylase inhibitor (IC_50_ = 42.32 ± 2.1 µM, against 78.24 ± 3.9 µM for acarbose; −8.5 kcal/mol). Over 200 ns of molecular dynamics, the **10**–α-amylase complex remained associated, with binding attributed by MM-GBSA mainly to van der Waals and lipophilic terms. **Conclusions**: Converting the dialdehyde into an enoate or a cyclic ether increases carbohydrase inhibition and determines which of the two enzymes is preferentially inhibited.

## 1. Introduction

Type 2 diabetes accounts for approximately 90% of all cases of diabetes mellitus and remains a major global health burden [1]. α-Amylase and α-glucosidase hydrolyze dietary carbohydrates into absorbable monosaccharides; inhibiting them delays glucose absorption and reduces postprandial glycemic excursions, an established approach to glycemic control [2,3]. Acarbose, the reference inhibitor in clinical use, is effective but frequently causes flatulence and abdominal discomfort, which limits adherence [4]. Inhibitors from natural sources with better tolerability therefore remain of interest.

Polygodial (**9**) is a drimane-type sesquiterpene dialdehyde and the principal constituent of *Drimys winteri* (Winteraceae), a tree native to Chile used by the Mapuche people for painful and inflammatory conditions [5,6]; leaves from Chilean populations contain a mean of 0.99% polygodial on a dry-weight basis, with marked variation between populations [7]. It also occurs in *Tasmannia lanceolata* [8], *Pseudowintera colorata* [9] and several *Polygonum* species [10]. Its reported pharmacology is broad antifungal action through membrane disruption and inhibition of mitochondrial ATP synthesis [11,12], anti-inflammatory [13], antinociceptive [14] and gastroprotective [8] effects, and blockade of the nociceptive sodium channels NaV1.7 and NaV1.8 [5] but its activity against α-amylase and α-glucosidase has not been reported.

**Figure 1:**
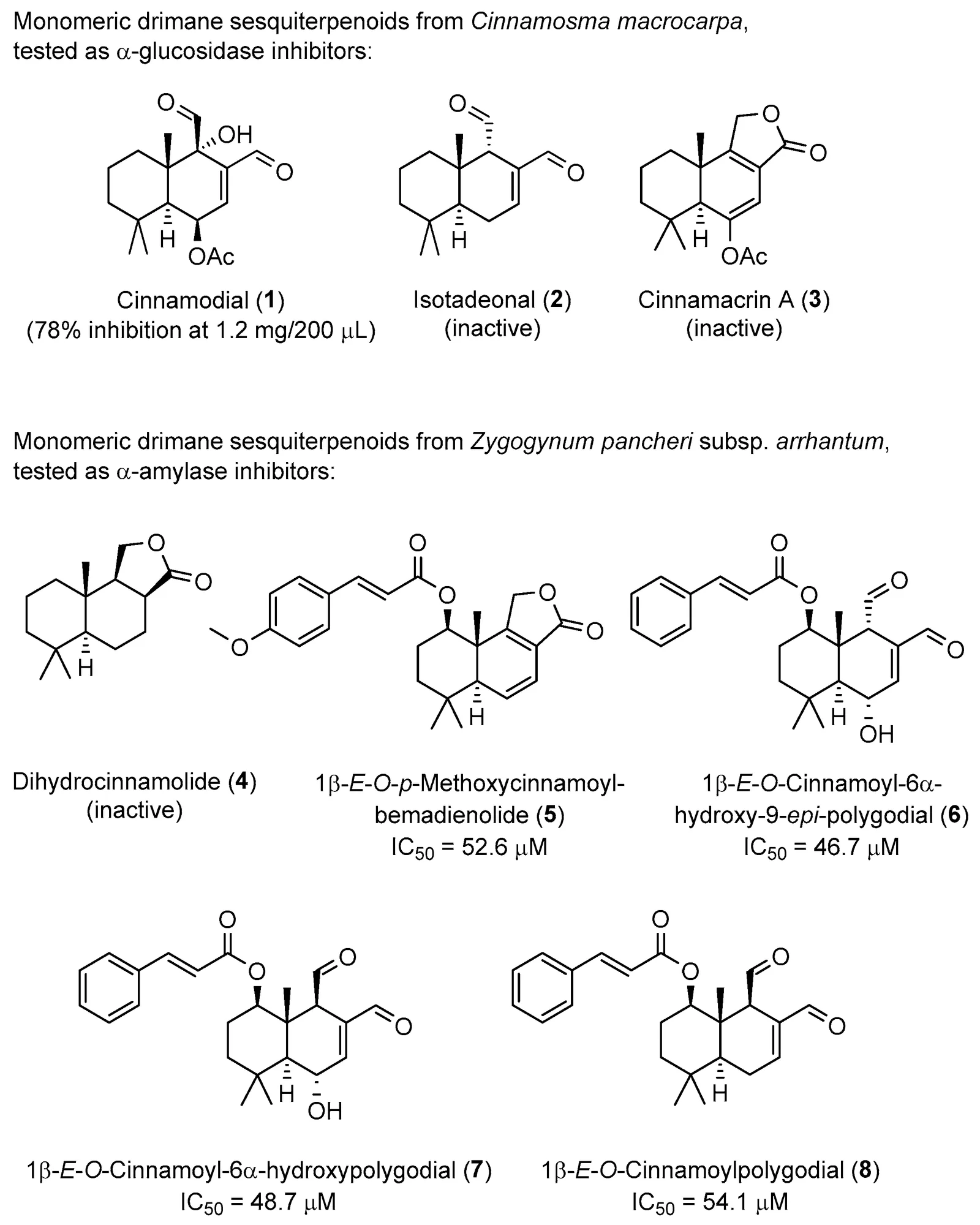
Monomeric drimane sesquiterpenoids previously evaluated against α-glucosidase (**1**–**3**, from *Cinnamosma macrocarpa*) and α-amylase (**4**–**8**, from *Zygogynum pancheri* subsp. *arrhantum*), with their reported activities. These compounds constitute the comparative baseline used in Section 4.

Evidence that the drimane skeleton can inhibit these two enzymes is scarce and is confined to the compounds shown in Figure 1. Among the monomeric drimanes from *Cinnamosma macrocarpa*, only cinnamodial (**1**) inhibited α-glucosidase, whereas isotadeonal (**2**) and cinnamacrin A (**3**) were inactive [15]; cinnamodial was also identified as an α-glucosidase inhibitor in *C. madagascariensis* [16]. From *Zygogynum pancheri* subsp. *arrhantum*, the drimanes **4**–**8** were tested against α-amylase in vitro: the cinnamoyl esters **5**–**8** gave IC_50_ values of 46.7–54.1 µM, while dihydrocinnamolide (**4**) was inactive [17], and **5** and **6** subsequently lowered fasting blood glucose in diabetic rats [17]. That isotadeonal (**2**), an unsubstituted drimane dialdehyde, was inactive indicates that the drimane core alone does not confer α-glucosidase inhibition and that peripheral modification is required. These eight compounds constitute the only quantitative baseline available for this chemotype and are used as such in Section 4.

Semisynthetic modification of polygodial is well precedented and alters its biological profile: its C9 epimer isotadeonal (**2**) inhibits the NF-κB pathway more strongly than the parent compound [6], and the enoates obtained by Wittig olefination at C12 are cytostatic toward drug-resistant cancer cells [18,19] and LNCaP prostate cancer cells [20], inhibit Na^+^/K^+^-ATPase with a preference for cerebral isoforms [21], and are active against *Trypanosoma cruzi* [22]. Whether this chemistry also modulates carbohydrase inhibition has not been examined. We therefore prepared the C12 enoate **10** and its two cyclization products **12a** and **12b**, measured their inhibition of α-glucosidase and α-amylase relative to acarbose, and used molecular docking, molecular dynamics simulation and MM-GBSA analysis to describe their binding.

## 2. Materials and Methods

Polygodial (**9**) was acquired from the natural product library of the Natural Products and Drug Discovery (NP&DD) Laboratory. The title compound was previously purified from *Drimys winteri* by column chromatography (CC) together with drimenol, isotadeonal and winterdial [23]. (Carbethoxymethylene) triphenylphosphorane (Ph_3_P=CHCO_2_Et), dry THF, sodium borohydride (NaBH_4_), and analytical thin-layer chromatography (TLC) 60F254 sheets were purchased from Sigma Aldrich (Darmstadt, Germany). Solvents for CC were purchased from Winkler (Santiago, Chile), distilled before use and dried over MgSO_4_.

### 2.1. Synthesis of Polygodial Derivative ***10***

A solution of polygodial (**9**, 234 g/mol, 500 mg, 2.14 mmol) in dry THF (30 mL) was placed in a vessel suited for microwave irradiation. Ph_3_P=CHCO_2_Et (350 mg, 1.00 mmol) was added dropwise, the vessel was sealed, placed in the microwave reactor, and irradiated at 160 °C for 10 min. After cooling, all volatiles were removed under reduced pressure, and the gummy residue was purified by column chromatography on silica with hexane/EtOAc (9:1 *v*/*v*) as eluent, affording compound **10** (304 g/mol, 560 mg, 86%) as a yellowish oil and as an inseparable 1.2:1.0 mixture of diastereomers. ^1^H-NMR data for the major diastereomer (9*R*)-**10** (obtained from the mixture) ^1^H NMR (600 MHz, CDCl_3_): *d* 9.46 (d, *J* = 4.8 Hz, 1H, H11), 7.32 (d, *J* = 16.3 Hz, 1H, H12), 6.50 (ddd, *J* = 6.1, 2.5, 2.5 Hz, 1H, H7), 5.49 (d, *J* = 16.3 Hz, 1H, H16), 4.20–4.13 (m, 2H, –OC*H*_2_CH_3_), 2.82 (m, 1H, H9), 2.31 (dm, *J* = 20.1 Hz, 1H, H6), 2.22 (m, 1H, H6), 1.83 (dq, *J* = 13.3, 2.6 Hz, 1H, H1), 1.64–1.56 (m, 2H, H2,3), 1.53–1.44 (m, 2H, H2,3), 1.33 (ddd, *J* = 17.1, 13.1, 4.2 Hz, 1H, H1), 1.26 (t, *J* = 7.2 Hz, 3H, –OCH_2_C*H*_3_), 1.19 (dd, *J* = 12.1, 4.5 Hz, 1H, H5), 1.00 (s, 3H), 0.93 (s, 3H), 0.89 (s, 3H). Residual ethyl acetate is present in the spectrum (δ ≈ 4.12 and 2.04 ppm, –OCH_2_– and –CH_3_ respectively), the latter showing an HMBC correlation to ~170 ppm; these signals overlap the ester –OCH_2_– region of **10** and account for the elevated integral there. NMR data for this diastereomer match those reported in the literature [24]. ^1^H-NMR data for the minor diastereomer (9*S*)-**10** (obtained from the mixture) ^1^H NMR (600 MHz, CDCl_3_): *d* 9.60 (d, *J* = 4.9 Hz, 1H, H11), 7.26 (d, *J* = 15.9 Hz, 1H, H12), 6.47 (dd, *J* = 5.0, 3.2 Hz, 1H, H7), 5.77 (d, *J* = 15.9 Hz, 1H, H16), 4.20–4.13 (m, 2H, –OC*H*_2_CH_3_), 2.75 (dd, *J* = 5.1, 1.0 Hz, 1H, H9), 2.49 (ddd, *J* = 20.2, 5.2, 5.2 Hz, 1H, H6), 2.18 (m, 1H, H6), 1.72 (dd, *J* = 11.8, 5.2 Hz, 1H H5), 1.64–1.56 (m, 2H, H2,3), 1.53–1.44 (m, 3H, H1,2,3), 1.27 (t, *J* = 7.3 Hz, 3H, –OCH_2_C*H*_3_), 1.22–1.16 (m, 1H, H1,2,3), 0.94 (s, 3H), 0.93 (s, 3H), 0.92 (s, 3H). ^13^C-NMR data of the diastereomeric mixture of (9*R*)-**10** and (9*S*)-**10**: ^13^C NMR (150 MHz, CDCl_3_) *d* 205.6, 202.1, 167.4, 167.2, 146.5, 146.1, 141.9, 141.7, 130.5, 129.0, 116.8, 116.6, 62.9, 62.5, 60.5, 60.5, 48.7, 44.7, 42.3, 41.9, 40.3, 37.8, 37.5, 36.8, 33.4, 33.2, 33.2, 32.9, 25.7, 25.0, 22.4, 22.0, 21.4, 18.6, 18.2, 15.7, 14.4, 14.4.

### 2.2. Reduction of Compounds ***10*** to Derivatives ***12a*** and ***12b***

A solution of **10** (304 g/mol, 420 mg, 1.38 mmol) in EtOH (50 mL) was stirred at room temperature, and NaBH_4_ (37.8 g/mol, 42 mg, 1.10 mmol) was added in portions. The reaction was stirred for 30 min., and then quenched with HCl (aq., 0.1 M, 10 mL). The mixture was extracted three times with EtOAc (50 mL). The solvents were removed under vacuum and the residue was purified by column chromatography using hexanes/MTBE mixture (7:3 *v*/*v*) to give **12a** (Rf: 0.63 hexane/EtOAc 4:1 *v*/*v*, 131 mg, 306 g/mol, 31%), and **12b** (Rf: 0.24 hexane/EtOAc 4:1 *v*/*v*, 170 mg, 322 g/mol, 38%).

### 2.3. NMR Analysis

The structural analysis was performed by NMR spectroscopy in CDCl_3_ using a Bruker Avance NEO 500 or 600 spectrometer (Bruker Biospin GmbH, Rheinstetten, Germany). NMR spectra were recorded at 600 or 500 MHz for ^1^H and 150 or 125 MHz for ^13^C, respectively. Signal assignments are based on the 2D-NMR experiments H, H-COSY, NOESY, HSQC, and HMBC.

### 2.4. α-Glucosidase Inhibition Assay

The α-glucosidase inhibitory activity was assessed using the protocol based on the breakdown principle of the p-nitrophenyl α-D-glucopyranoside (pNPG), as described by Yurtci and Ergene with minor modification [25]. In this experiment, stock solutions were prepared in DMSO at 100 mM and diluted two-fold in DMSO to give the working series. One microliter of each was added per well, giving final compound concentrations of 15.5–500 µM in a 201 µL reaction volume and a constant DMSO concentration of 0.5% (*v*/*v*). To each well, 40 µL of α-glucosidase (0.5 U/mL) from *Saccharomyces cerevisiae* (Sigma-Aldrich, catalog no. G5003) was then added. Control wells received the same volume of DMSO. Then 120 µL phosphate buffer (0.1 M with pH 7.4) was added to each well. Following a five-minute incubation period, 40 µL of substrate solution (5 mM) was added in each well, which was then incubated again at 37 °C for 30 min. To stop the reaction, 30 µL of Na_2_CO_3_ (0.1 M) was added. The absorbance of *p*-nitrophenol was measured at 405 nm using VICTOR Nivo microplate reader. Acarbose (from Sigma Aldrich, catalog no.A8980) was employed as positive control in this experiment. The inhibitory percentage was calculated using the following Formula (1):

(1) Inhibition% = [(A_control − A_sample)/A_control] × 100

### 2.5. α-Amylase Inhibition Assay

In the α-amylase inhibition assay, the starch iodine protocol was used with minor modification [26,27]. In a 96-well plate, the reaction mixture containing 50 μL of phosphate buffer (100 mM, pH 6.8), 10 μL of porcine pancreatic α-amylase enzyme (2 U/mL) from Sigma-Aldrich (catalog no. A3176), and 20 μL of varying concentrations (15.5 µM, 31.25 µM, 62.5 µM, 125 µM, 250 µM and 500 µM) of polygodial and its derivatives was pre-incubated at 37 °C for 20 min. Compounds were diluted from the 100 mM DMSO stock into assay buffer, and 20 µL was added to a final reaction volume of 100 µL, giving final concentrations of 15.5–500 µM and a constant DMSO concentration of 0.5% (*v*/*v*). Control wells received the same proportion of DMSO. Then, 20 μL of 1% soluble starch (100 mM phosphate buffer pH 6.8) was added as a substrate and incubated at 37 °C for 30 min. Next, lugol (100 µL) and HCl (0.1 M, 25 µL) solutions were added to complete the reaction. The absorbances were then recorded at 630 nm and the results were expressed as the half-maximal inhibitory concentration (IC_50_). The reference compound used was acarbose, as already used in αglucosidase assays. The inhibitory percentage was calculated using Formula (1).

### 2.6. Statistical Analysis

Each assay was run in triplicate as three independent experiments, and values are given as the mean ± standard deviation. IC_50_ values were obtained by fitting the percentage-inhibition data to sigmoidal dose–response curves in GraphPad Prism (version 10), which was also used for the plots. The agreement between the in vitro potencies and the docking scores was quantified per enzyme across the four drimane compounds using the Spearman rank correlation (IC_50_ versus docking score) and the Pearson correlation (log_10_ IC_50_ versus docking score).

### 2.7. Molecular Docking Protocol

Polygodial and its three derivatives were docked against human pancreatic α-amylase (1HNY) and the C-terminal catalytic subunit of human maltase–glucoamylase (3TOP) using AutoDock Vina 1.1.2 through the PyRx interface, following the protocol we have applied previously to carbohydrase targets [28]. For each receptor a single grid box was defined manually and centered on the substrate-binding pocket, with a grid spacing of 0.375 Å. For α-amylase (1HNY) the box was centered on the catalytic residues Asp197, Glu233 and Asp300 at (x, y, z) = (10.44, 48.92, 17.61) Å with dimensions 25 × 30 × 25 Å. For α-glucosidase (3TOP) the box was centered on the co-crystallized acarbose of the retained chain at (x, y, z) = (−31.79, 34.81, 26.36) Å with dimensions 25 × 26 × 25 Å. Each box was sized to enclose the catalytic residues while allowing the ligands to sample the full pocket.

### 2.8. Ligand Preparation

The four NMR-characterized compounds served as ligands. Structures were drawn in ChemDraw 12.0 and exported as .sdf files (https://cambridgesoft-chemdraw-ultra.software.informer.com/12.0/; accessed 1 December 2025), generated from SMILES with stereochemistry defined explicitly; both C9 epimers of **10** and both C12 epimers of **12a** were built and docked. Geometries were energy-minimized prior to docking [29]. Compound **10** was represented by its major (9*R*) diastereomer and **12a** by its major (12*R*) diastereomer, these being the predominant species in the 1.2:1 and 4:1 mixture isolated from the synthesis.

### 2.9. Retrieval and Preparation of Receptor Proteins

Crystal structures of the two targets were taken from the Protein Data Bank (https://www.rcsb.org; accessed 5 December 2025): human pancreatic α-amylase (1HNY) and the C-terminal catalytic subunit of human maltase–glucoamylase (3TOP). The same three criteria governed both choices: a human enzyme, a complete catalytic domain, and a resolution suitable for docking and not whether a ligand was bound. 1HNY, solved at 1.80 Å, keeps the structural calcium and the neighboring chloride ion that the α-amylase active site requires for integrity [30], 3TOP represents the subunit that hydrolyses the longer maltose oligomers [31], its bound acarbose additionally enabled the redocking validation in Section 2.10. The pairing of these two receptors for this same set of targets follows recent work on natural-product carbohydrase inhibitors [32].

### 2.10. Docking Protocol Validation: Redocking Procedure

The docking protocol was validated by redocking the co-crystallized inhibitor into its own receptor. Acarbose was extracted from the α-glucosidase structure (3TOP), prepared by the same procedure applied to the test compounds, and redocked into the receptor from which it had been removed, using the same grid and search parameters. Agreement with the experimental pose was assessed as the heavy-atom root-mean-square deviation between the top-ranked docked pose and the crystallographic coordinates, calculated in the crystal reference frame without superposition. No equivalent test was possible for α-amylase, since the structure used (1HNY) is the unliganded form and contains no co-crystallized ligand.

### 2.11. Protein–Ligand Interaction Analysis

Binding of each ligand within the prepared active sites was computed in PyRx 0.8 [33], and the resulting poses were rendered and the contacts assigned in BIOVIA Discovery Studio Visualizer 2021 [34].

### 2.12. Molecular Dynamics Simulation Protocol

Three selected docked complexes were subjected to molecular dynamics simulations using Desmond [35] (Schrödinger v2019-4 ) with the OPLS3e force field. Three systems were simulated: derivative **10** with α-amylase (1HNY), derivative **12a** with α-amylase, and derivative **12a** with α-glucosidase (3TOP).

Ligands were prepared in LigPrep from stereochemically defined SMILES, with ionization states set at pH 7.0 ± 2.0, and each complex was processed in the Protein Preparation Wizard (bond-order assignment, addition of hydrogens, optimization of the hydrogen-bond network, and restrained minimization to a heavy-atom RMSD of 0.30 Å). Each complex was solvated with TIP3P water in an orthorhombic box with a 10 Å buffer, neutralized, and adjusted to 0.15 M NaCl; the **10**–α-amylase system comprised 20,353 atoms (4208 waters, 14 Na^+^, 12 Cl^−^), with the remaining compositions in Table S1.

Systems were relaxed using the default Desmond protocol Brownian dynamics in the NVT ensemble at 10 K, successive NVT and NPT stages at 10 K and 300 K with the solute heavy-atom restraints progressively released, and with harmonic positional restraints initially set to 50 kcal mol^−1^ Å^−2^ and progressively released, followed by a final unrestrained NPT stage. Production runs of 200 ns were propagated in the NPT ensemble at 300 K and 1.01325 bar using a Nosé–Hoover chain thermostat and a Martyna–Tobias–Klein barostat, with a RESPA integrator (2.0 fs bonded and short-range, 6.0 fs long-range), smooth particle-mesh Ewald electrostatics (tolerance 1 × 10^−9^) and a 9.0 Å short-range cutoff. Coordinates were saved every 100 ps (~2000 frames per trajectory).

Trajectories were analyzed with the Schrödinger Simulation Interaction Diagram tools, extracting protein Cα RMSD, per-residue RMSF, protein–ligand contact occupancies and ligand solvent-accessible surface area; ligand RMSD is reported fitted on the protein (displacement within the binding site), with the own-reference value (internal flexibility) noted where relevant. Principal component analysis and dynamic cross-correlation maps were computed on the Cα coordinates, and binding free energies were estimated by MM-GBSA (Prime) over 41 frames sampled at 5 ns intervals (mean ± SD).

## 3. Results

### 3.1. Synthesis and Structure Analysis of Polygodial Derivatives

**Scheme 1:**
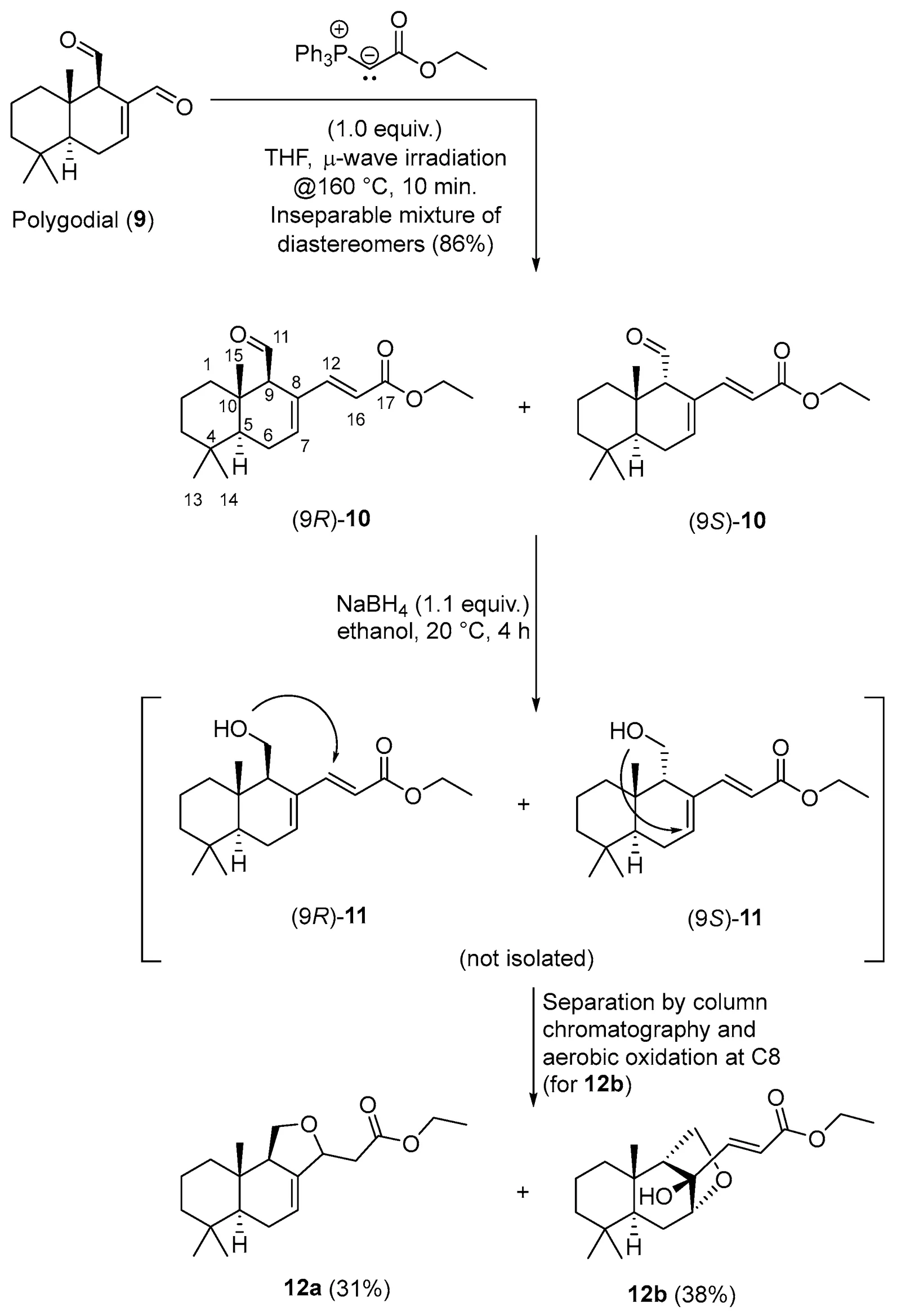
Synthesis of polygodial derivatives by regioselective Wittig olefination followed by reduction with sodium borohydride.

The Wittig olefination of polygodial (**9**) with stabilized ylides has previously been reported to furnish mono-enoate **10** with high regioselectivity. In contrast to previous reports, however, we used microwave-accelerated reaction conditions at 160 °C and could thus obtain the desired compound **10** in very short reaction times of just 10 min in a high yield of 86%. In spite of the high reaction temperature, the olefination is nevertheless highly regioselective and occurs exclusively at the sterically less hindered aldehyde at position C12. However, it was noted that epimerization at C9 occurs, which resulted in the formation of **10** as an inseparable 1.2:1.0 mixture of (9*R*)- and (9*S*)-diastereomers (Scheme 1).

Chemoselective reduction in the remaining aldehyde group at C11 was accomplished using NaBH_4_ in ethanol. The expected diastereomeric primary alcohols **11** were not observed, but two cyclization products of this intermediate could be isolated: an annellated tricyclic tetrahydrofuran **12a**, resulting from a conjugate 1,4-addition of the OH-group to the enoate was isolated in 31% yield as an inseparable 4:1 mixture diastereomers, and a bridged tricyclic product **12b** was isolated as a single diastereomer in 38% yield. The latter product is the result of a conjugate 1,6-addition of the OH-group to the dienoate and, apparently, a subsequent aerobic oxidation of the allylic methine carbon C8. Structural assignments of cyclization products **12a** and **12b** were accomplished by 1D- and 2D-NMR methods. The NMR data and the positional assignments are listed in Table 1.

### 3.2. In Vitro Enzyme Inhibition

**Figure 2:**
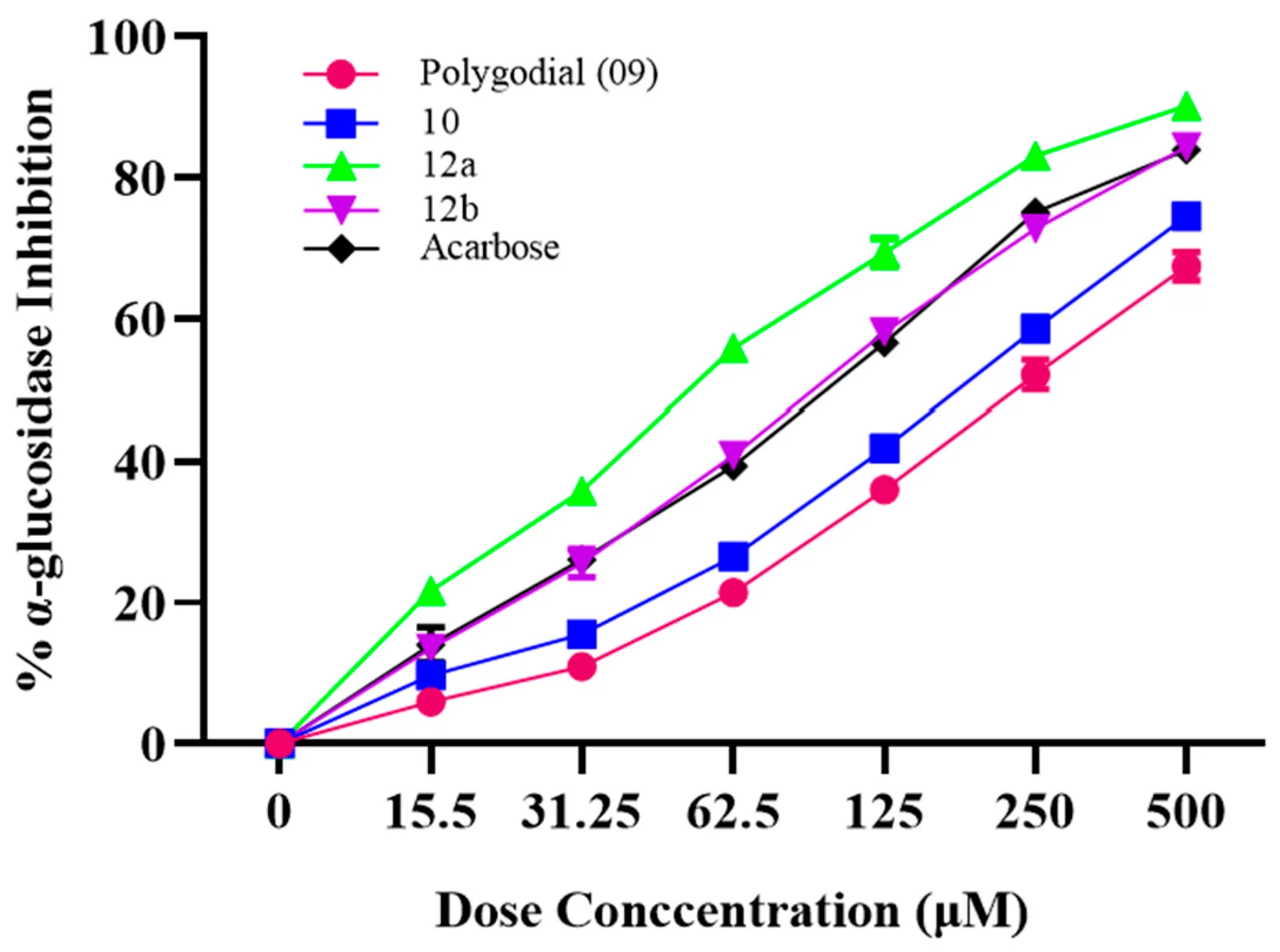
Concentration-dependent inhibition of α-glucosidaseby polygodial and its derivatives along with acarbose as a positive control.

**Figure 3:**
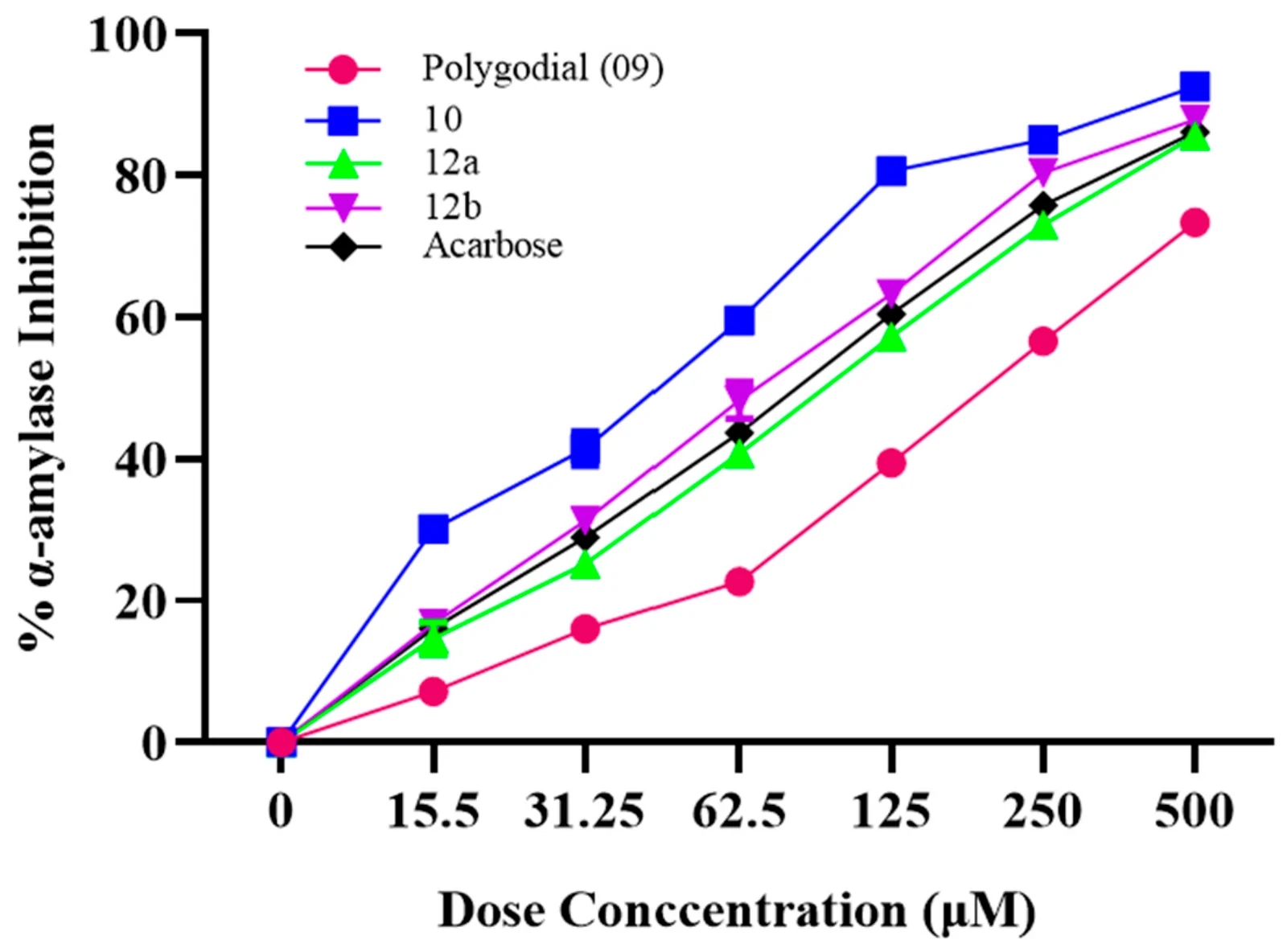
Concentration-dependent inhibition of α-amylase by polygodial and its derivatives along with acarbose as a positive control.

The inhibitory effects of polygodial (**9**) and its three semisynthetic derivatives **10**, **12a** and **12b** against α-glucosidase and α-amylase were evaluated at concentrations ranging from 15.5 to 500 µM. Acarbose, a clinically used antidiabetic drug, served as the positive control. The IC_50_ values are presented in Table 2, while the dose-dependent inhibition curves are illustrated in Figure 2 and Figure 3.

All tested compounds demonstrated concentration-dependent inhibition of α-glucosidase activity (Figure 2). Among the derivatives, **12a** exhibited the strongest α-glucosidase inhibitory activity with an IC_50_ value of 53.98 ± 3.0 µM, which was lower than that of acarbose (IC_50_ = 90.36 ± 4.0 µM). Compound **12b** showed comparable activity to acarbose with an IC_50_ of 91.33 ± 1.7 µM. Compound **10** displayed moderate inhibition (IC_50_ = 168.01 ± 1.0 µM), while the parent compound polygodial (**9**) showed the weakest activity among all tested compounds with an IC_50_ of 224.11 ± 2.0 µM (Table 2).

At the highest concentration tested (500 µM), **12a** achieved approximately 90% inhibition, followed by **12b** and acarbose with inhibition rates around 85%. Compound **10** and polygodial (**9**) reached maximum inhibition values of approximately 85% and 68%, respectively.

The α-amylase inhibitory activity of the test compounds also followed a dose-dependent pattern (Figure 3). Compound **10** demonstrated the strongest α-amylase inhibition with an IC_50_ value of 42.32 ± 2.1 µM, which was notably lower than that of acarbose (IC_50_ = 78.24 ± 3.9 µM). Compound **12b** also showed better activity than the standard drug, with an IC_50_ of 67.84 ± 2.44 µM. Compound **12a** exhibited an IC_50_ of 88.21 ± 1.91 µM, indicating slightly weaker activity compared to acarbose. The parent compound polygodial showed the lowest α-amylase inhibitory activity with an IC_50_ of 198 ± 1.88 µM (Table 2).

At 500 µM concentration, **10** achieved approximately 93% inhibition of α-amylase activity, followed by **12b** and acarbose at around 86–87%. Compound **12a** showed approximately 85% inhibition, while polygodial reached about 75% inhibition at the same concentration.

### 3.3. Molecular Docking Results

#### 3.3.1. Virtual Screening and Binding Affinity Analysis

To further investigate the molecular basis of enzyme inhibition, molecular docking studies were performed for polygodial (**9**) and its three derivatives **10**, **12a** and **12b** against α-amylase (PDB ID: 1HNY) and α-glucosidase (PDB ID: 3TOP). The binding affinities and interacting amino acid residues are summarized in Table 3.

#### 3.3.2. α-Amylase Docking Analysis

**Figure 4:**
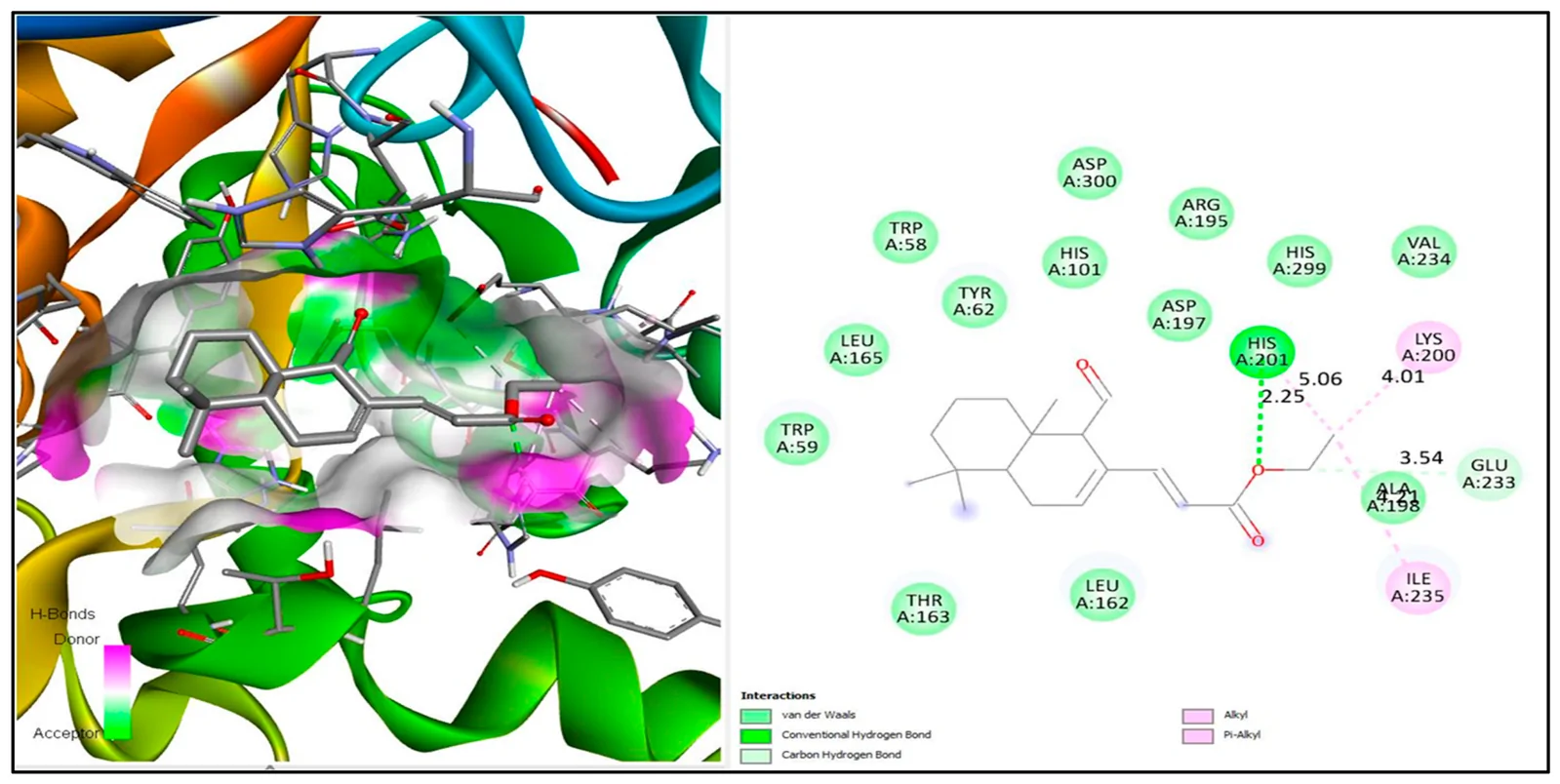
Two-dimensional interaction diagram and three-dimensional binding pose of compound **10** in the active site of α-amylase (PDB 1HNY).

Among the tested compounds, **10** exhibited the highest binding affinity against α-amylase with a score of −8.5 kcal/mol (Table 3). The two-dimensional interaction analysis revealed that **10** formed conventional hydrogen bonds with HisA:201 (2.25 Å) and GluA:233 (3.54 Å). π–alkyl interactions were observed with LysA:200 (4.01 Å) and IleA:235, while van der Waals contacts were present with TrpA:59 and surrounding residues, contributing to the stability of the ligand–receptor complex (Figure 4).

Compound **12b** demonstrated the second-highest binding affinity of −8.0 kcal/mol against α-amylase. The docking analysis showed that **12b** formed a conventional hydrogen bond with GluA:233 and carbon–hydrogen bonds with AlaA:198 (3.52 Å and 4.05 Å). π–alkyl interactions were observed with LysA:200 (5.25 Å) and HisA:201, along with alkyl contacts with IleA:235. Van der Waals interactions were present with TrpA:59, TrpA:58, AspA:300, and HisA:305 (Figure S18C).

Compound **12a** showed a binding affinity of −7.4 kcal/mol with α-amylase. The interaction pattern revealed predominantly hydrophobic interactions, with a π–alkyl contact with TrpA:59 (5.35 Å) and an alkyl interaction with LeuA:165. Van der Waals contacts were observed with GlnA:63 (3.97 Å), TyrA:62, ThrA:163, and other surrounding residues (Figure S18B).

The parent compound polygodial exhibited the lowest binding affinity of −6.8 kcal/mol among the tested compounds. Polygodial formed a conventional hydrogen bond with GlnA:63 (3.10 Å), along with van der Waals interactions with TrpA:59, TrpA:58, AspA:300, and HisA:305 (Figure S18A). The relatively weaker binding affinity of polygodial compared to its derivatives suggests that structural modifications enhanced the enzyme–ligand interactions.

Given that **12a** demonstrated significant in vitro α-amylase inhibitory activity (IC_50_ = 88.21 ± 1.91 µM) despite its moderate binding affinity (−7.4 kcal/mol), we investigated an alternative conformer of this compound to better understand its interaction profile. The **12a**-conformer exhibited a binding affinity of −6.8 kcal/mol against α-amylase, forming a conventional hydrogen bond with TyrA:62. Van der Waals interactions were observed with GlnA:63, TrpA:59, and surrounding residues. The lower binding energy of this conformer compared to the initial pose suggests conformational flexibility that may influence the compound’s biological activity. This observation indicates that **12a** may adopt multiple binding orientations within the enzyme active site, and the in vitro activity likely represents the cumulative effect of these conformational states.

#### 3.3.3. α-Glucosidase Interaction Analysis

**Figure 5:**
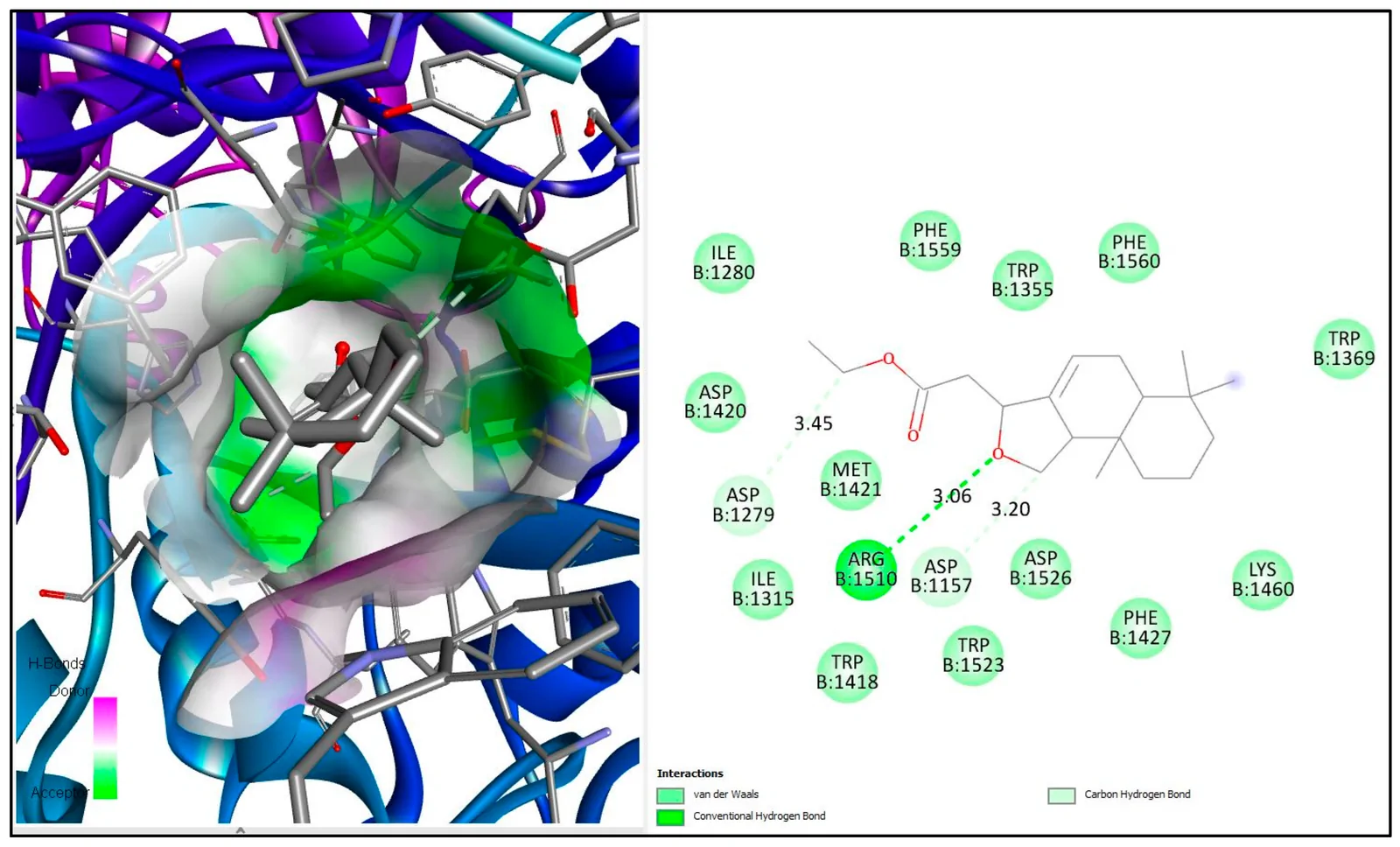
Two-dimensional interaction diagram and three-dimensional binding pose of compound **12a** in the active site of α-glucosidase (PDB 3TOP).

For α-glucosidase, **12a** demonstrated the strongest binding affinity with a score of −8.1 kcal/mol (Table 3). The interaction analysis revealed that **12a** formed conventional hydrogen bonds with AspB:1279 and AspB:1157 (3.20 Å), along with a carbon–hydrogen bond with MetB:1421 (3.06 Å). Van der Waals interactions were observed with ArgB:1510, IleB:1280, AspB:1420 (3.45 Å), TrpB:1355, and multiple surrounding residues, contributing to the overall binding stability (Figure 5).

To further explore the binding characteristics of **12a**, conformational analysis was performed. The **12a** conformer exhibited a binding affinity of −7.4 kcal/mol against α-glucosidase. The interaction analysis revealed conventional hydrogen bonds with TrpB:1148 (3.06 Å) and ThrB:1150. π–alkyl interactions were observed with PheB:995, along with van der Waals contacts with surrounding residues. Although this conformer displayed lower binding affinity compared to the primary pose (−8.1 kcal/mol), the presence of multiple binding orientations suggests that the observed in vitro activity of **12a** against α-glucosidase may result from dynamic equilibrium between different conformational states within the active site.

Compound **12b** exhibited a binding affinity of −7.3 kcal/mol against α-glucosidase. The compound formed a conventional hydrogen bond with AsnB:1404 (3.01 Å) and a carbon–hydrogen bond with LeuB:1401 (3.96 Å). π–alkyl interactions were observed with LeuB:1412 (4.65 Å and 5.19 Å) and TyrB:1282, while alkyl contacts were present with ProB:1329 (4.76 Å). Van der Waals interactions with GluB:1400, PheB:1289 (3.66 Å), and surrounding residues further stabilized the complex (Figure S19C).

Compound **10** showed a binding affinity of −6.7 kcal/mol with α-glucosidase. The interaction pattern revealed a conventional hydrogen bond with TrpB:1148 (3.06 Å), along with π–σ interaction at 5.08 Å. Alkyl interactions were observed with AlaB:973 (3.84 Å), and π–alkyl contacts with PheB:995. Van der Waals interactions were present with CysB:996, GlyB:992, and other surrounding residues (Figure S19B).

Polygodial exhibited the lowest binding affinity of −6.0 kcal/mol against α-glucosidase. The compound formed conventional hydrogen bonds with ThrA:1150 (3.80 Å) and ArgA:1206. Van der Waals interactions were observed with ThrA:1196 (3.04 Å and 6.25 Å), HisA:1149, GlyA:992, and other surrounding residues (Figure S19A).

Superposition with the crystallographic acarbose (Figure S21) shows that, within the shared binding pocket, **12a** alone reproduces the acarbose contacts, its ring oxygen and ester group lying within 3 Å of Asp1157, Asp1279 and Arg1510. The poses of polygodial, **10** and **12b** are displaced from these residues and do not reach the catalytic center. That the compound engaging the substrate-binding residues most closely is also the most active α-glucosidase inhibitor of the series is consistent with, though it does not establish, direct competition with substrate binding.

#### 3.3.4. Redocking Performance and Pose Reproduction

Redocking of acarbose into 3TOP reproduced the crystallographic binding mode with a heavy-atom RMSD of 1.45 Å for the top-ranked pose, below the 2.0 Å value conventionally taken to indicate a successful redocking. Of the 44 heavy atoms, 37 lay within 2 Å of their crystallographic positions and 19 within 1 Å, with the residual deviation confined to the terminal glucose unit (median per-atom deviation 1.07 Å; maximum 3.55 Å). The redocked pose recovered the interactions observed in the crystal structure, including contacts with Asp1157, Asp1279, Asp1420, Asp1526, Trp1355, Trp1369, His1584 and Thr1586 (Figure S20). Given that acarbose contains 22 rotatable bonds, this level of agreement indicates that the protocol reproduces the experimental binding mode for this receptor.

### 3.4. Dynamic Stability of Selected Complexes

Molecular dynamics simulations of 200 ns were performed to examine whether the docked complexes persist under solvated conditions. Compounds **10** and **12a** were simulated with α-amylase, allowing the two derivatives to be compared directly in the same enzyme, and **12a** was also simulated with α-glucosidase.

#### 3.4.1. Conformational Stability

**Figure 6:**
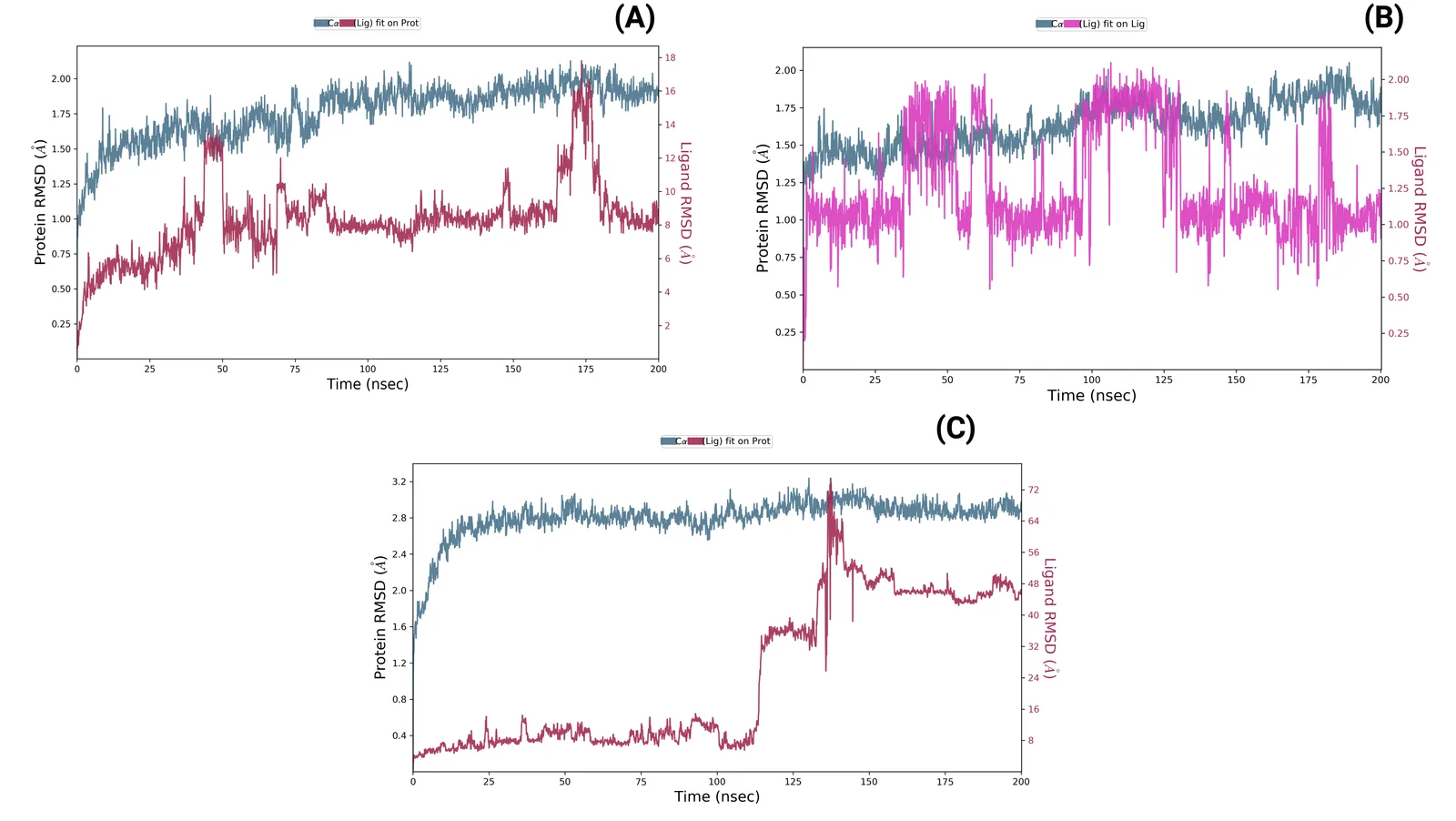
Root-mean-square deviation over the 200 ns trajectories. (**A**) **10**–α-amylase, (**B**) **12a**–α-amylase and (**C**) **12a**–α-glucosidase.

Both α-amylase complexes equilibrated within the first 40 ns and remained stable thereafter. For **10**, the protein Cα RMSD plateaued at 1.90 ± 0.08 Å over the final 100 ns and the ligand adopted a defined position within the cleft, with an internal conformational RMSD of 1.14 ± 0.21 Å (Figure 6A). The **12a** complex behaved similarly, with a protein Cα RMSD of 1.75 ± 0.10 Å and a ligand internal RMSD of 1.32 ± 0.38 Å (Figure 6B). Ligand solvent exposure was essentially constant for 10 (173 → 171 Å^2^) and decreased slightly for **12a** (192 → 168 Å^2^), indicating that neither ligand became progressively solvated.

In contrast, **12a** did not remain bound to α-glucosidase. Starting from the docked pose in the substrate-binding site, the ligand remained in place for approximately 110 ns and then left the site, its displacement relative to the protein rising above 40 Å for the remainder of the trajectory (Figure 6C). The residues contacted in the second half of the run share no overlap with those contacted initially, and the ligand solvent-accessible surface area increased from 219 to 315 Å^2^. An independent simulation started from a second docked pose gave the same outcome. This complex is therefore not analyzed further, and its implications are considered in Section 4.

#### 3.4.2. Residue Flexibility

**Figure 7:**
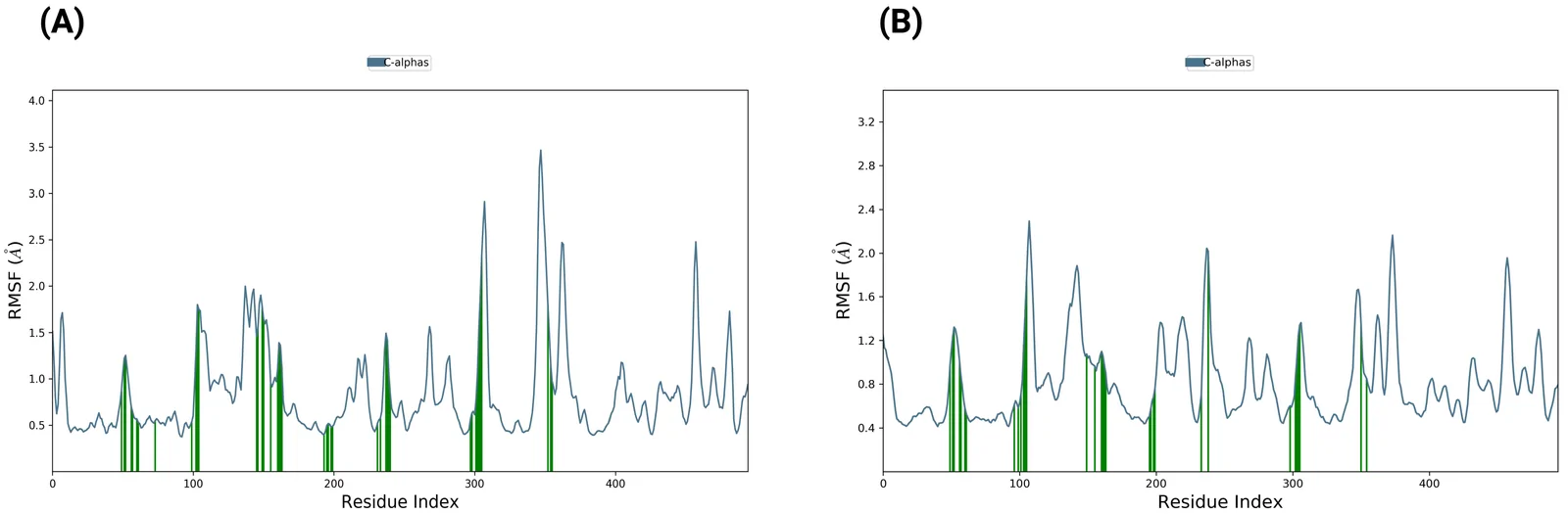
Per-residue root-mean-square fluctuation of the Cα atoms for (**A**) **10**–α-amylase and (**B**) **12a**–α-amylase. Green markers indicate residues in contact with the ligand.

RMSF profiles showed that no large residue fluctuations were observed in either ligand-bound α-amylase complex. Residues in contact with the ligand fluctuated by 1.08 Å (**10**) and 1.00 Å (**12a**), against protein-wide means of 0.85 and 0.82 Å, with the largest fluctuations confined to surface loops and chain termini (Figure 7).

#### 3.4.3. Protein–Ligand Contacts

**Figure 8:**
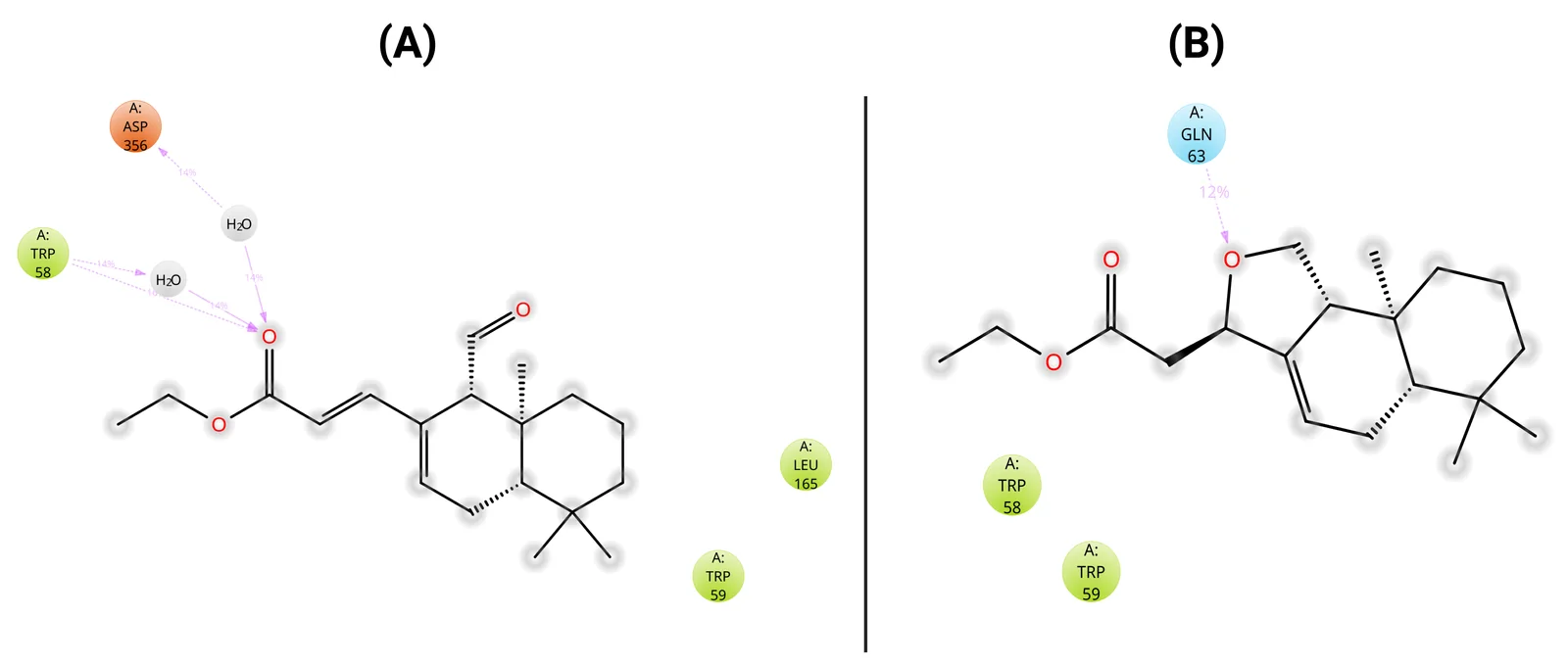
Representative post-simulation two-dimensional interaction diagrams for (**A**) **10** with α-amylase and (**B**) **12a** with α-amylase. Water-mediated hydrogen bonds are indicated by H_2_O.

Both derivatives engage the aromatic surface formed by Trp58 and Trp59 (Figure 8). For **10**, Trp59 was contacted in 30% of frames and Trp58 in 22%, the latter through a direct hydrogen bond, with further contributions from Tyr151 (12%), Leu165 (12%) and Tyr62 (10%), and a water-mediated contact to Asp356 (13%). For **12a**, Trp59 dominated (49%) alongside Trp58 (23%), and the principal polar anchor was a hydrogen bond to Gln63 present in 18% of frames, supported by water bridges to Thr163 and Gln63. The two derivatives therefore occupy the same region of the cleft but differed in their polar anchors: a direct hydrogen bond to Trp58 for **10** and a hydrogen bond to Gln63 for **12a**.

**Figure 9:**
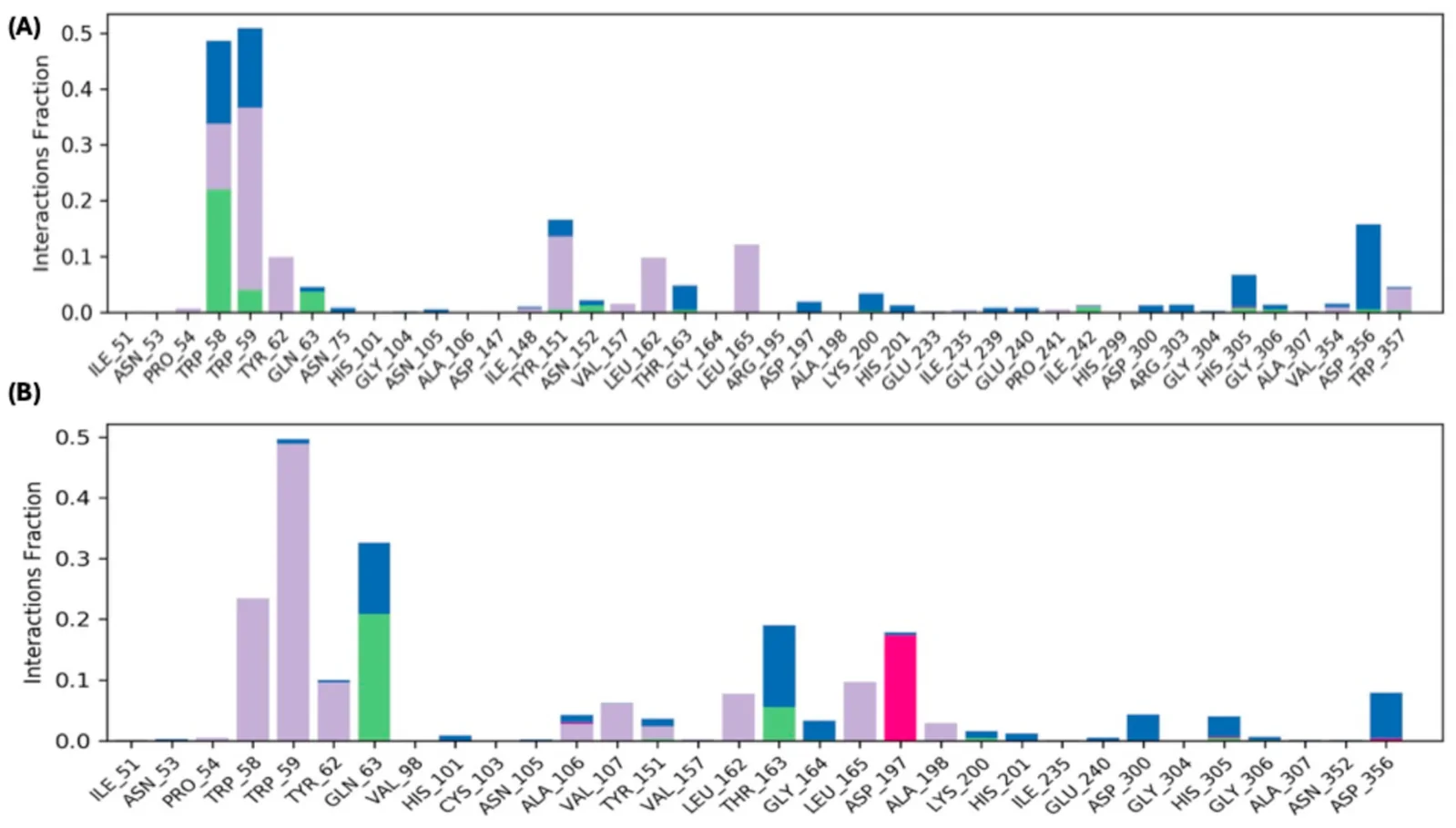
Protein–ligand contact histograms over 200 ns for (**A**) **10**–α-amylase and (**B**) **12a**–α-amylase, showing the fraction of the trajectory in which each residue is engaged. Interaction types are distinguished by color: hydrogen bonds (green), hydrophobic contacts (purple), ionic interactions (magenta) and water bridges (blue). Numerical occupancies are given in Table S4.

In both complexes the contacts established during the first 40 ns persisted to the end of the trajectory. The interactions identified by docking were partially retained: the direct hydrogen bond to His201 seen in the docked pose of **10** was replaced during the simulation by a water-mediated network involving Trp58 and Asp356 (Figure 9).

#### 3.4.4. Collective Motions

**Figure 10:**
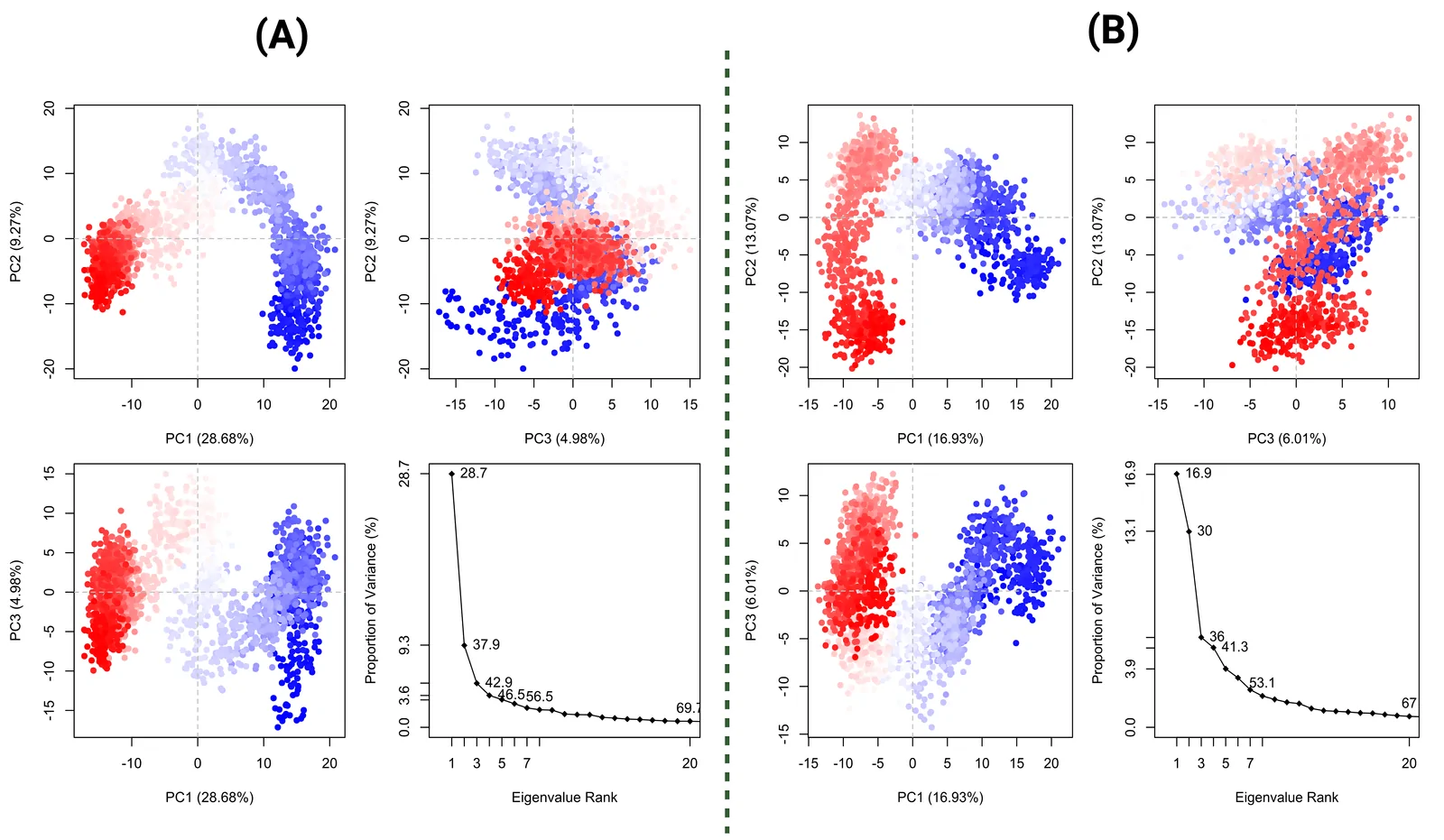
Principal component analysis of the Cα coordinates for (**A**) **10**–α-amylase and (**B**) **12a**–α-amylase, showing projections onto PC1–PC3 with the corresponding eigenvalue scree plots. Each point represents one saved trajectory frame, colored by simulation time from the earliest frames (dark blue) through intermediate frames (pale) to the latest (dark red). The colors indicate temporal progression and do not encode energy, interaction strength or residue identity.

**Figure 11:**
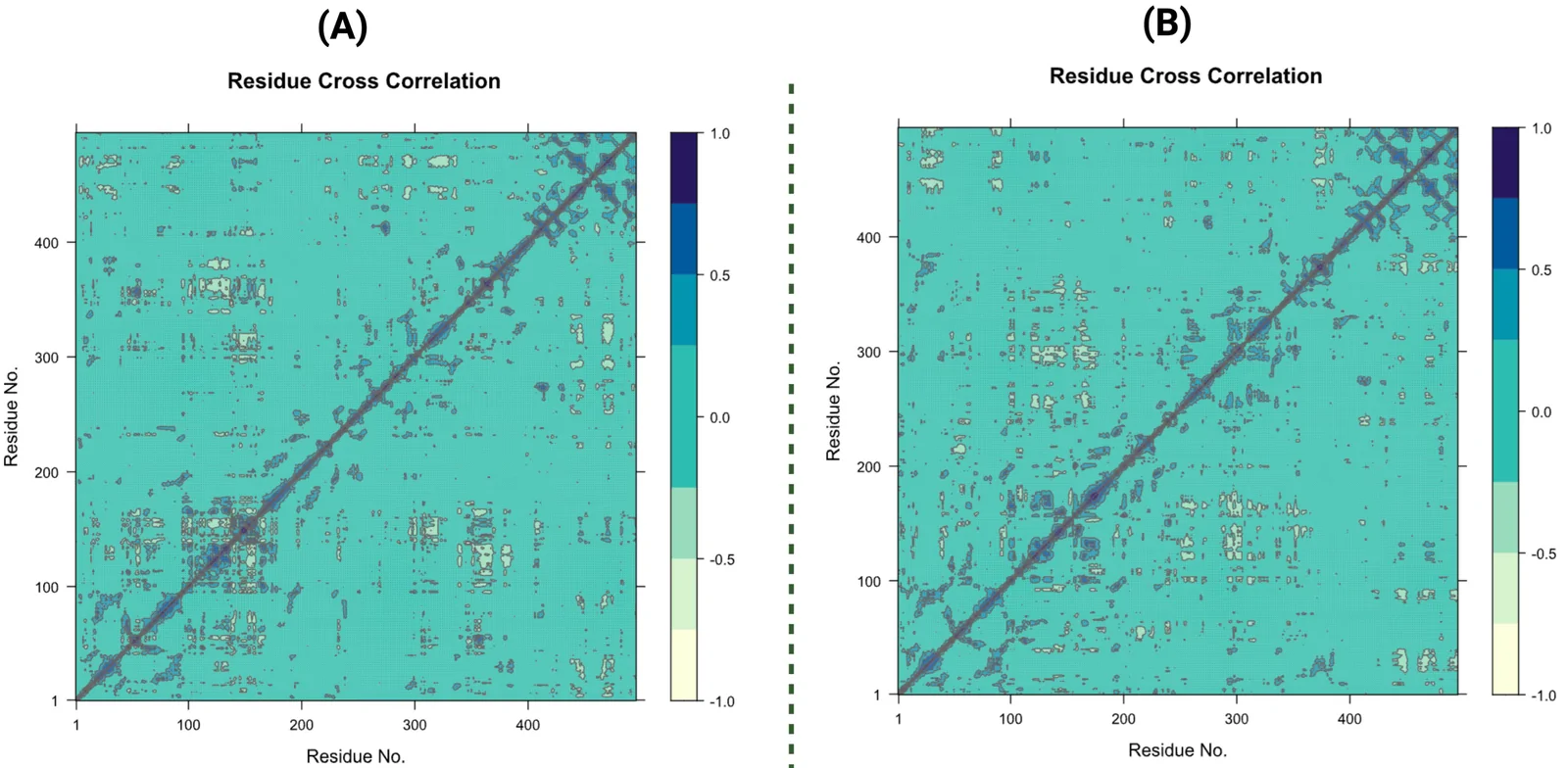
Dynamic cross-correlation maps of the Cα atoms for (**A**) **10**–α-amylase and (**B**) **12a**–α-amylase. The scale runs from +1 (dark blue, residues moving in the same direction) through 0 (turquoise, uncorrelated) to −1 (pale yellow, anticorrelated). The dark diagonal reflects the self-correlation of each residue.

Principal component analysis and dynamic cross-correlation maps were computed on the Cα coordinates of the two α-amylase complexes, both of which remained associated throughout their trajectories. The motion was low-dimensional in both systems: for **10**–α-amylase the first component accounted for 28.7% of the total variance and the first three for 42.9%, while for **12a**–α-amylase the corresponding values were 16.9% and 36.0% (Figure 10). The more even distribution of variance across components in the **12a** complex indicates that its motion is less dominated by a single collective mode. The cross-correlation maps of both complexes are dominated by short-range positive correlations along the diagonal, with limited long-range coupling between distant regions (Figure 11).

The projections show a time-ordered distribution of frames, with early and late frames occupying distinguishable regions of the landscape. We interpret this conservatively. Such a progression is consistent with relaxation from the docked starting structure, but a smooth temporal ordering of principal components can also arise from incomplete sampling rather than from a genuine conformational transition, since the leading components of diffusive motion on a flat energy surface approach cosine functions [36]. Cosine content and essential subspace overlap between trajectory halves were not computed here, so the separation is described rather than interpreted as evidence of conformational stabilization. Matched simulations of the free enzymes were likewise not performed, and no inference is therefore drawn regarding the influence of ligand binding on correlated motion. The **12a**–α-glucosidase trajectory was excluded from this analysis, since the ligand left the binding site during the simulation (Section 3.4.1) and its later frames no longer describe a protein–ligand complex.

The two α-amylase complexes present comparable conformational landscapes. In both, the variance is distributed across many components rather than concentrated in one: approximately seven components are required to account for half the total fluctuation in each system, and the first three capture 42.9% for **10** and 36.0% for **12a**. A diffuse essential subspace of this kind is consistent with the architecture of pancreatic α-amylase, whose catalytic domain is a (β/α)_8_ barrel lacking the interdomain hinges that generate dominant low-frequency modes in multidomain proteins. The collective motion sampled here is therefore local, arising principally from surface loops and chain termini, as the RMSF profiles also indicate (Section 3.4.2). Neither ligand induces a large-scale collective motion, which is consistent with both compounds binding within a preformed cleft rather than driving a conformational rearrangement.

The modest difference between the two systems, a larger first component for **10**, indicates that its trajectory is somewhat more directional, but we do not attach mechanistic weight to this. The ligand-level descriptors do not follow the same ordering: **12a** in fact showed the smaller variation in position relative to the protein (7.46 ± 1.39 Å against 9.10 ± 1.98 Å for **10**), so protein collective motion and ligand positional stability report on different properties of the complex and need not correlate. Comparison with the α-glucosidase system was not attempted, both because the ligand dissociated during that trajectory and because variance fractions from proteins of different size and flexibility are not directly comparable.

#### 3.4.5. Binding Free Energy

End-state binding free energies were estimated by MM-GBSA for the **10**–α-amylase complex over 41 frames sampled at 5 ns intervals (Table 4). The mean ΔG_bind was −58.18 ± 11.08 kcal/mol, and −61.89 ± 10.51 kcal/mol over the final 100 ns. The decomposition was dominated by van der Waals (−32.74 ± 5.23) and lipophilic (−36.94 ± 7.49) terms, with a small Coulombic contribution (−4.47 ± 4.14) opposed by a polar solvation penalty (+14.55 ± 3.21). Ligand strain remained low throughout (2.24 ± 0.97 kcal/mol). Values obtained with this method are large in absolute terms and are used here descriptively rather than as absolute affinities.

## 4. Discussion

Polygodial (**9**) was the weakest inhibitor of both enzymes tested (IC_50_ = 224.11 ± 2.0 µM against α-glucosidase and 198.00 ± 1.88 µM against α-amylase), and every modification of its dialdehyde improved potency. This is consistent with the only comparable literature datum: isotadeonal (**2**), the C9 epimer of polygodial and likewise an unsubstituted drimane dialdehyde, was inactive against α-glucosidase among the monomeric sesquiterpenoids of *Cinnamosma macrocarpa* [15] (Figure 1). Taken together, these observations indicate that the drimane dialdehyde motif is not itself a carbohydrase pharmacophore, and that activity in this scaffold is determined by what is appended to it.

The two modifications diverge in which enzyme they favor. Olefination at C12 (**10**) improved α-amylase inhibition 4.7-fold relative to **9** (198.00 → 42.32 µM) but α-glucosidase inhibition only 1.3-fold (224.11 → 168.01 µM). Cyclisation of the same enoate to the annellated tetrahydrofuran **12a** produced the opposite bias: a 4.2-fold gain against α-glucosidase (224.11 → 53.98 µM) against a 2.2-fold gain at α-amylase. The bridged ether **12b**, formed by 1,6- rather than 1,4-addition, lies between the two and inhibits both enzymes to a similar extent (91.33 ± 1.7 and 67.84 ± 2.44 µM). Selectivity across this small series is therefore governed by the geometry of the C11–C12 region rather than by the decalin core, which is common to all four compounds.

Set against the compounds in Figure 1, which represent the whole of the reported evidence for monomeric drimanes at these two targets, the present derivatives are competitive but not exceptional. The cinnamoyl esters **5**–**8** from *Zygogynum pancheri* subsp. *arrhantum* inhibit α-amylase with IC_50_ values of 46.7–54.1 µM [17]; 10 falls in the same range (42.32 ± 2.1 µM) while carrying a small C12 enoate in place of a C1 aryl ester that is, it reaches comparable potency at lower molecular weight and without the bulky C1 cinnamoyl ester motif. For α-glucosidase no numerical comparison is possible, since cinnamodial (**1**), the only monomeric drimane previously reported as active, was described by percentage inhibition at a single high dose rather than by an IC_50_ [15]. Within the limits of that literature, **12a** (53.98 ± 3.0 µM) appears to be the first monomeric drimane for which a defined micromolar IC_50_ against α-glucosidase has been determined. Assay formats, enzyme sources and substrate concentrations differ between these studies, so the comparisons are indicative and are not a substitute for side-by-side testing.

The docking scores reproduced the experimental ranking in both enzymes without exception. For α-amylase the order was **10** > **12b** > **12a** > polygodial, and for α-glucosidase **12a** > **12b** > **10** > polygodial, in each case matching the order of the measured IC_50_ values (Table 2). Expressed numerically, the Spearman rank correlation between IC_50_ and docking score was +1.00 for both enzymes, and the Pearson correlation between log_10_ IC_50_ and docking score was +0.979 (R^2^ = 0.96) for α-amylase and +0.989 (R^2^ = 0.98) for α-glucosidase. Including acarbose as a fifth point lowered these values to ρ = +0.90 and r = +0.885 and +0.951, respectively, which is expected given that acarbose belongs to a different chemotype. These coefficients should be read as descriptive. With four compounds, the exact two-sided probability of obtaining a perfect rank agreement by chance is 0.083, so the correlation, although complete, is not statistically demonstrable at conventional thresholds; the same caution applies to the Pearson values, which rest on two degrees of freedom. What the analysis supports is that the scoring function orders this small congeneric series consistently with the experiment in both targets, which is the level of agreement docking is expected to provide.

The two structures differ in ligation state, and since receptor conformation can influence docking outcomes, we assessed the magnitude of this difference for α-amylase. Superposition of 1HNY with an acarbose-bound pancreatic α-amylase (1OSE) [37] gives a global Cα RMSD of 0.41 Å over 496 aligned residues, with 86.7% sequence identity; sixteen of the seventeen residues lining the substrate cleft are identical, and their Cα positions differ by 0.05–0.46 Å. The principal difference lies in the loop spanning residues 304–307, where Gly306 and His305 are displaced by 4.6 and 2.0 Å. In the present results this region contributes van der Waals contacts only, and no compound formed a hydrogen bond within it, so the conformational difference is peripheral to the poses obtained. The unliganded structure therefore represents the substrate cleft in a form closely comparable to the inhibitor-bound state. We note nonetheless that rigid-receptor docking does not model induced fit, and that conformational adaptation may contribute to the measured potencies in ways not captured here.

Acarbose behaves differently from the drimanes in both receptors. It forms five conventional hydrogen bonds in each, more than any test compound, yet its docking scores are intermediate (−7.1 and −6.9 kcal/mol). This is consistent with its polysaccharide-like structure, in which affinity arises from many individually weak polar contacts, whereas the drimane derivatives combine a lipophilic decalin core with one or two directional hydrogen bonds. The MM-GBSA decomposition supports the same picture: binding of **10** to α-amylase is dominated by van der Waals and lipophilic terms, with only a small electrostatic contribution.

The simulations add a dynamic dimension to this comparison. Both **10** and **12a** remained associated with α-amylase over 200 ns and settled onto the same aromatic surface formed by Trp58 and Trp59, but differed in their polar anchor: a direct hydrogen bond to Trp58 for **10**, and a hydrogen bond to Gln63 for **12a**. The docked pose of **10** was not retained unchanged: the direct hydrogen bond to His201 was replaced during the simulation by a water-mediated network involving Trp58 and Asp356. This reorganization is a common outcome when rigid docking poses are relaxed in explicit solvent, and it illustrates why static scores are best treated as a ranking device rather than as a description of the bound state.

Three limitations bear on how these results should be read. First, **12a** did not form a persistent complex with the α-glucosidase structure over 200 ns in two independent simulations started from different docked poses. The result is chemically consistent: the maltase–glucoamylase site is polar and adapted to hydroxyl-rich carbohydrates, whereas the present compounds are lipophilic drimane ethers with few hydrogen-bond donors. The α-glucosidase findings therefore rest on the in vitro measurements and the docking analysis, and the dynamic characterization is presented for α-amylase, where both derivatives could be compared in the same enzyme.

Second, the α-glucosidase inhibition reported here was measured against the Saccharomyces cerevisiae enzyme, whereas the structure available for modeling is the human protein. The docking therefore describes how a drimane ether can be accommodated in a carbohydrase active site rather than modeling the assay system itself, and the comparison with acarbose describes relative behavior within this specific assay. Acarbose is a markedly weaker inhibitor of the yeast enzyme than of mammalian intestinal α-glucosidases, so the fold-differences reported here should not be extrapolated to human enzymes or to clinical efficacy. Third, the mode of inhibition has not been established, no selectivity against related glycosidases was assessed, and no cellular or in vivo data are presented. Enzyme-kinetic characterization of **10** and **12a** against the mammalian enzymes, together with expansion of the series around the C11–C12 ring, are the logical next steps.

## 5. Conclusions

In this work we examined the antidiabetic potential of polygodial and three of its semisynthetic derivatives and found that converting the reactive dialdehyde of the parent sesquiterpene into a conjugated enoate (**10**) or into cyclic ether scaffolds (**12a**, **12b**) changes both the potency and the enzyme selectivity of carbohydrate hydrolase inhibition. The annellated tetrahydrofuran **12a** was the preferred α-glucosidase inhibitor, whereas the C12 enoate **10** was the more effective α-amylase inhibitor, each more active than acarbose against its respective target under the present in vitro assay conditions, while **12b** retained activity toward both enzymes. This divergence indicates that the position and type of modification on the drimane skeleton, rather than the scaffold alone, determines which enzyme is engaged most strongly. Taken together, these results identify polygodial-derived enoates and cyclic ethers as tractable starting points for selective or dual carbohydrase inhibition and define enzyme-kinetic and in vivo work as the necessary next step.

## Figures and Tables

**Table 1:**
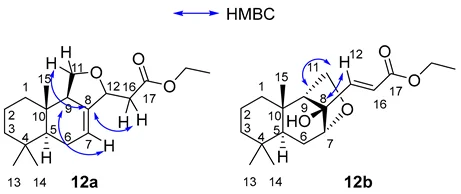
NMR data and positional assignment of compounds **12a** and **12b**.

Position	12a		12b	
	*δ*_H_ (mult., J/Hz) ^a^	*δ*_C _(Type of C) ^b^	*δ*_H _(mult., J/Hz) ^a^	*δ*_C_ (Type of C) ^b^
1	1.53 (m) 1.16 (m)	40.7 (CH_2_)	1.44 (m) 1.31 (dm, 11.7)	36.2 (CH_2_)
2	1.53 (m) 1.43 (m)	18.7 (CH_2_)	1.45 (m) 1.42 (m)	18.4 (CH_2_)
3	1.42 (m) 1.21 (m)	42.3 (CH_2_)	1.45 (m) 1.15 (ddd, 13.9, 13.9, 3.9)	42.3 (CH_2_)
4	--	32.8 (C_q_)	--	32.9
5	1.29 (dd, 12.0, 5.2)	50.0 (CH)	1.45 (m)	38.8
6	2.16 (m) 1.90 (m)	24.1 (CH_2_)	2.09 (ddd, 15.3, 4.5, 1.3) 1.73 (ddd, 15.3, 12.9, 2.5)	23.3 (CH_2_)
7	5.42 (m)	116.5 (=CH)	3.21 (m)	63.7 (CH)
8	--	140.9 (=C_q_)	--	60.7 (C_q_)
9	2.48 (m)	55.3 (CH)	1.86 (dd, 6.8, 4.8)	50.2 (CH)
10	--	34.1 (C_q_)	--	34.9 (C_q_)
11	3.87 (dd, 8.7, 8.7) 3.55 (dd, 9.6, 8.7)	66.5 (CH_2_)	3.85 (dd, 11.4, 4.8) 3.77 (dd, 11.4, 6.8)	61.5 (CH_2_)
12	4.74 (m)	75.8 (CH)	6.86 (d, 15.7)	150.4 (=CH-)
13	0.86 (s)	33.4 (CH_3_)	0.87 (s)	23.0 (CH_3_)
14	0.90 (s)	27.6 (CH_3_)	0.85 (s)	33.1 (CH_3_)
15	0.75 (s)	13.4 (CH_3_)	0.97 (s)	22.7 (CH_3_)
16	2.52 (dd, 15.5, 4.2) 2.41 (dd, 15.5, 8.6)	39.8 (CH_2_)	5.99 (d, 15.7)	121.1 (=CH-)
17	--	171.5 (C=O)	--	166.3 (C=O)
–O*CH*_2_CH_3_	4.16 (q, 7.2)	60.7 (CH_2_)	4.17 (q, 7.1)	60.7 (CH_2_)
–OCH_2_*CH_3_*	1.26 (t, 7.2)	14.3 (CH_3_)	1.27 (t, 7.1)	14.3 (CH_3_)

^a 1^H NMR (500 MHz, CDCl_3_). ^b 13^C NMR (125 MHz, CDCl_3_).

**Table 2:** The IC_50_ values of polygodial and its derivatives for α-glucosidase and α-amylase.

Compound	α-Glucosidase (µM)	α-Amylase (µM)
Polygodial (**9**)	224.11 ± 2.0	198.00 ± 1.88
**10**	168.01 ± 1.0	42.32 ± 2.1
**12a**	53.98 ± 3.0	88.21 ± 1.91
**12b**	91.33 ± 1.7	67.84 ± 2.44
Acarbose	90.36 ± 4.0	78.24 ± 3.9

**Table 3:** Binding affinities of polygodial and its derivatives against α-amylase (1HNY) and α-glucosidase (3TOP). Full lists of interacting residues are given in Table S4.

Ligand	α-Amylase ΔG (kcal/mol)	H-Bonds	α-Glucosidase ΔG (kcal/mol)	H-Bonds
Polygodial (**9**)	−6.8	1	−6.0	3
**10**	−8.5	1	−6.7	1
**12a**	−7.4	0	−8.1	1
**12b**	−8.0	1	−7.3	1
Acarbose	−7.1	5	−6.9	5

Values correspond to the major diastereomer of each compound. H-bonds = number of conventional hydrogen bonds in the top-ranked pose.

**Table 4:** MM-GBSA binding free energy and energy decomposition for the **10**–α-amylase complex (kcal/mol), averaged over 41 frames sampled at 5 ns intervals across the 200 ns trajectory (mean ± SD).

System (Ligand/Target)	ΔG_bind	ΔG_vdW	ΔG_lipo	ΔG_Coul	ΔG_solv,GB
10/1HNY (α-amylase)	−58.18 ± 11.08	−32.74 ± 5.23	−36.94 ± 7.49	−4.47 ± 4.14	+14.55 ± 3.21

ΔG_vdW, van der Waals; ΔG_lipo, lipophilic; ΔG_Coul, Coulomb; ΔG_solv,GB, polar (generalized Born) solvation. Over the final 100 ns, ΔG_bind = −61.89 ± 10.51 kcal/mol; mean ligand strain energy 2.24 ± 0.97 kcal/mol. Values obtained with this method are large in absolute terms and are used descriptively rather than as absolute affinities.

## Data Availability

The underlying data are available from the published article and the accompanying Supporting Information File (pdf, copies of all NMR spectra). FAIR data (primary NMR-FID files for compounds **10**, **12a**, and **12b**) and the Supporting Information File are openly available through the Zenodo-data repository at https://doi.org/10.5281/zenodo.18875481.

## Supplementary Materials

The following supporting information can be downloaded at: https://www.mdpi.com/article/10.3390/pharmaceutics18080960/s1.
