# Supplementary material for: Semisynthetic Derivatives of Polygodial as α-Glucosidase and α-Amylase Inhibitors: In Vitro Evaluation, Molecular Docking and Molecular Dynamics Simulation
Source: Pharmaceutics. 2026 Aug 5;18(8):960. doi: 10.3390/pharmaceutics18080960 (PMC13516052; doi:10.3390/pharmaceutics18080960)
Supplement: Supplementary file 1 [file pharmaceutics-18-00960-s001.zip › pharmaceutics-4401178-supplementary.pdf]

SUPPORTING INFORMATION

Semisynthetic derivatives of Polygodial as α-glucosidase and α-amylase inhibitors: In vitro evaluation, molecular docking and molecular dynamics simulation

Viviana Burgos[1], Cecilia Villegas[2], Carlos Sanzana[3], Benjamin Oporto[3], Bernd schmidt[4], Vaderament-A. Nchiozem ngnitedem[4], Muhammad Javid Iqbal[3]* and Cristian Paz [3]*

[1] Escuela de Tecnología Médica, Facultad de Salud, Universidad Santo Tomás, Temuco 4780000,
Chile. vburgos7@santotomas.cl (V.B)
[2] Departamento de Ciencias Biológicas y Químicas, Facultad de Recursos Naturales, Universidad
Católica de Temuco, Rudecindo Ortega, Temuco 4780000, Chile. cecilia.villegas@uct.cl (C.V)
[3]Laboratory of Natural Products & Drug Discovery, Center CEBIM, Department of Basic Sciences,
Faculty of Medicine, Universidad de La Frontera, Temuco 4780000, Chile; c.sanzana02@ufromail.cl (C.S); b.oporto02@ufromail.cl (B.O)
[4]Institut für Chemie, Universität Potsdam, Karl-Liebknecht-Str. 24-25, D-14476 Potsdam, Germany. bernd.schmidt@uni-potsdam.de (B.S.); n.vaderamentalexe@gmail.com (V-A.N.)
* Correspondence: m.iqbal01@ufromail.cl (M.J.I); cristian.paz@ufrontera.cl; Tel.: +56 45 259 2825 (C.P.)

*Correspondence: m.iqbal01@ufromail.cl, cristian.paz@ufrontera.cl; Tel.: +56 45 259 2825.

Contents:

Copies of NMR-spectra for compounds **10**, **12a** and **12b**

Figure S1: ^{1}H NMR (600 MHz, $CDCl_3$) of **10**.

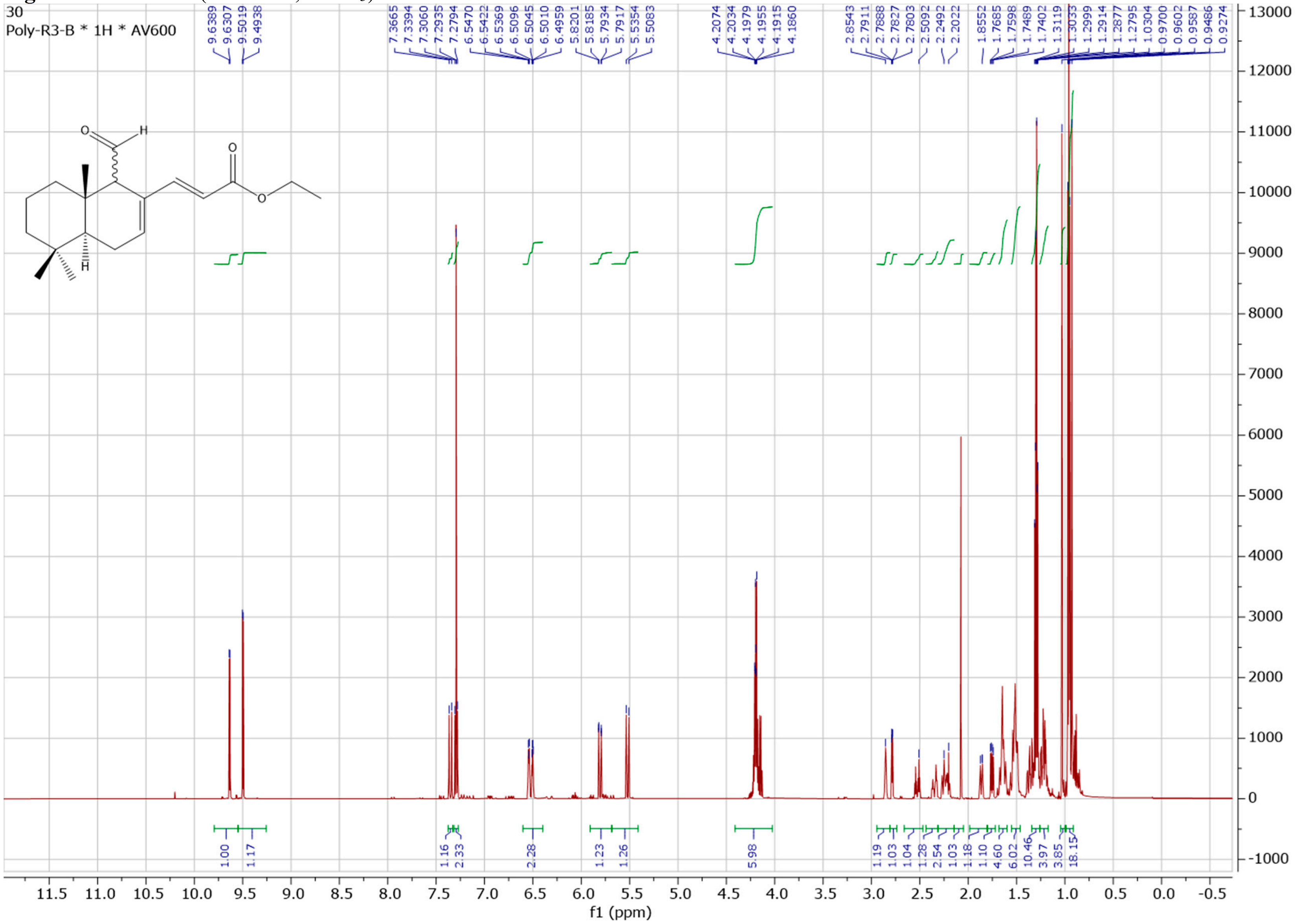

Figure S2: $^{13}C\{^{1}H\}$ NMR (150 MHz, $CDCl_3$) of **10**.

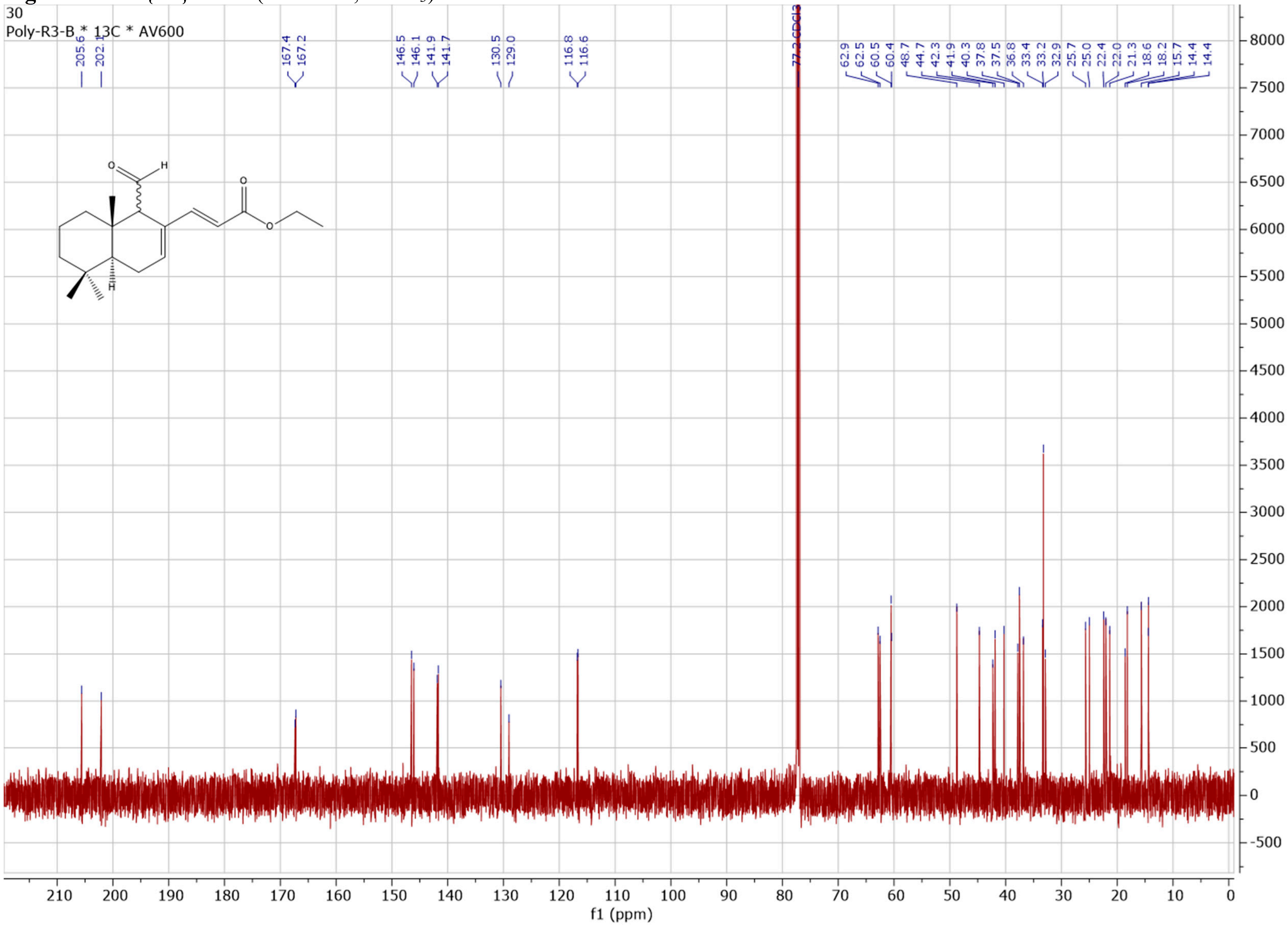

Figure S3: H, H-COSY (600 MHz, $CDCl_3$) of **10**.

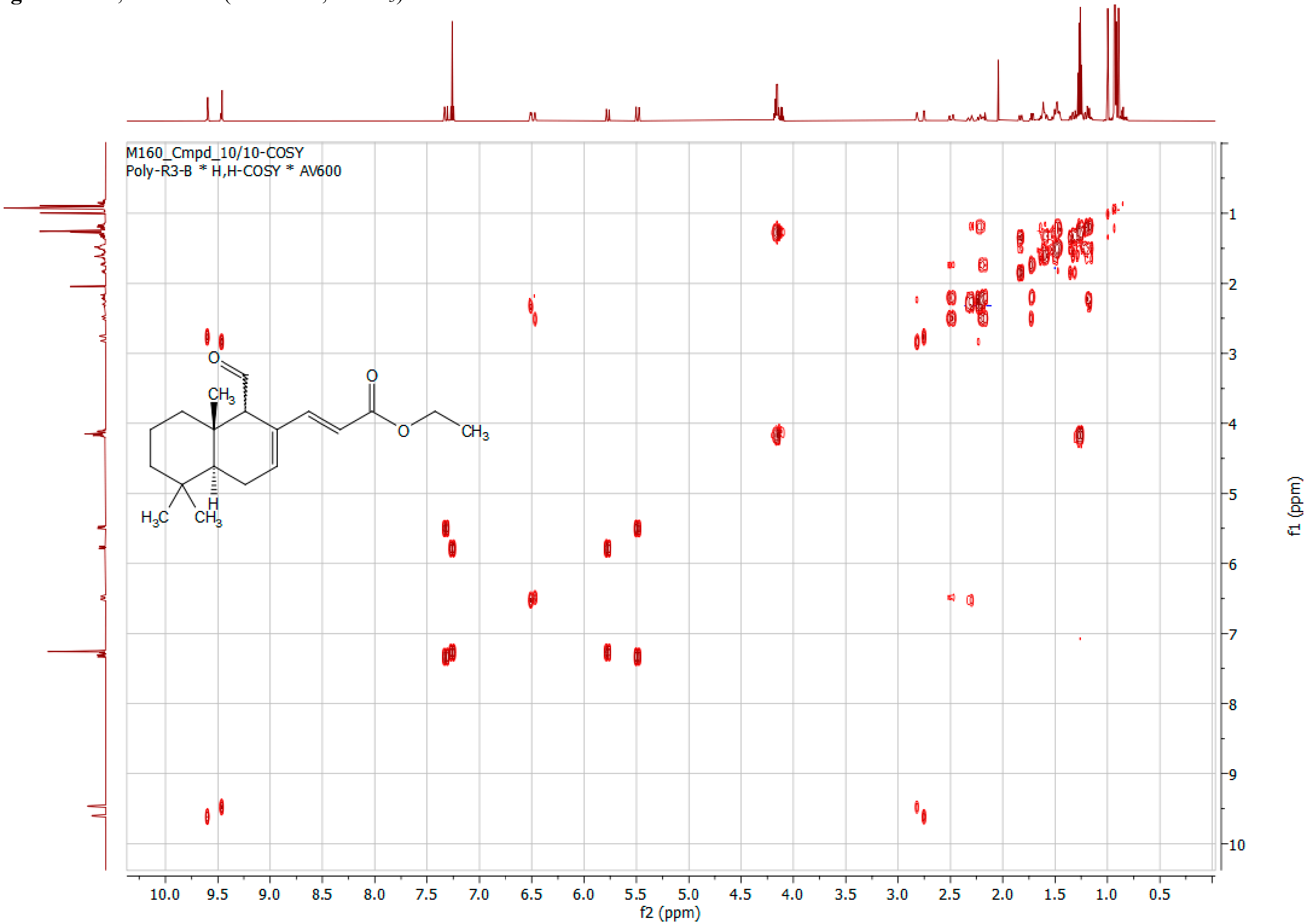

Figure S4: HSQC (600/150 MHz, $CDCl_3$) of **10**.

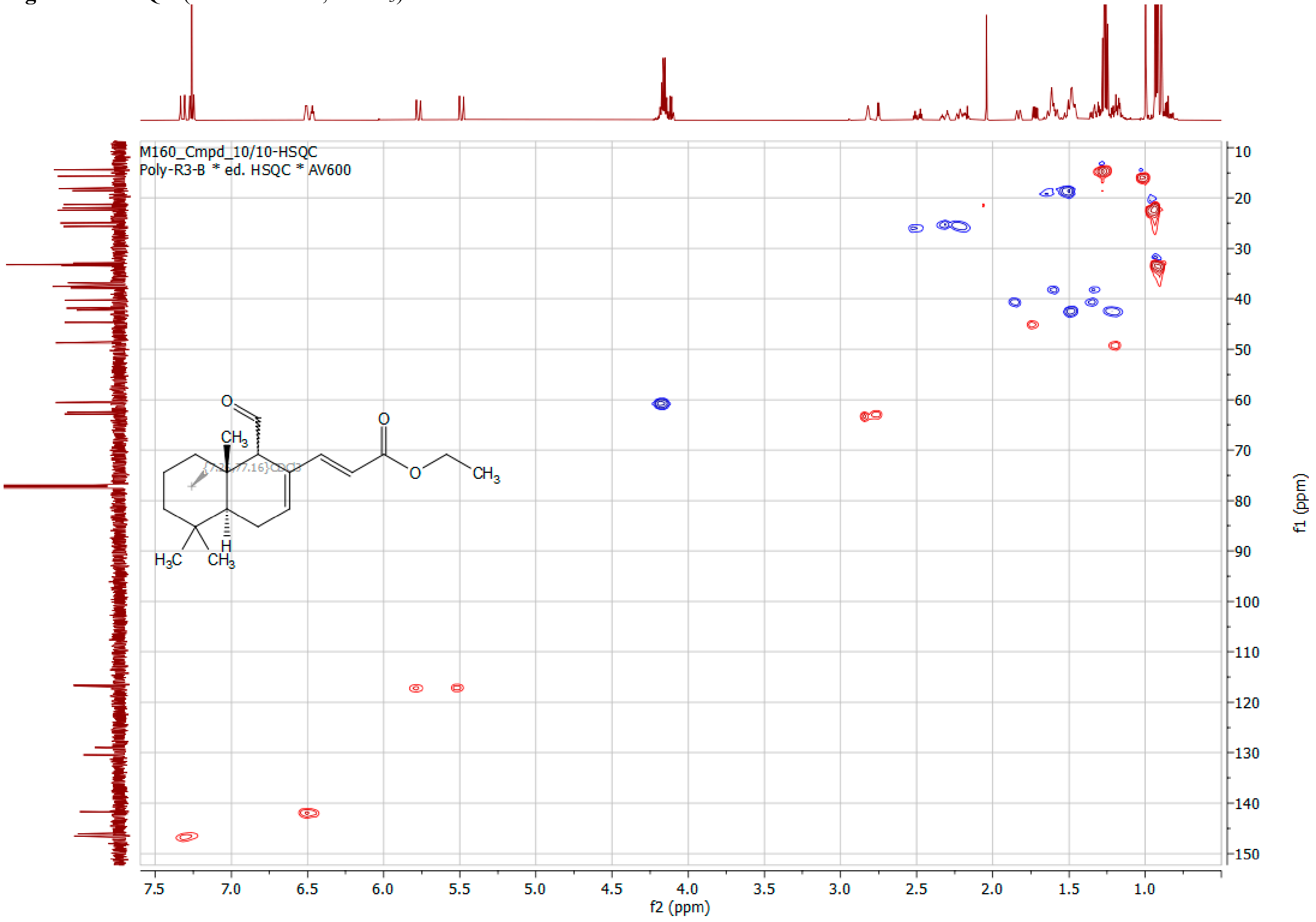

Figure S5: HMBC (600/150 MHz, $CDCl_3$) of **10**.

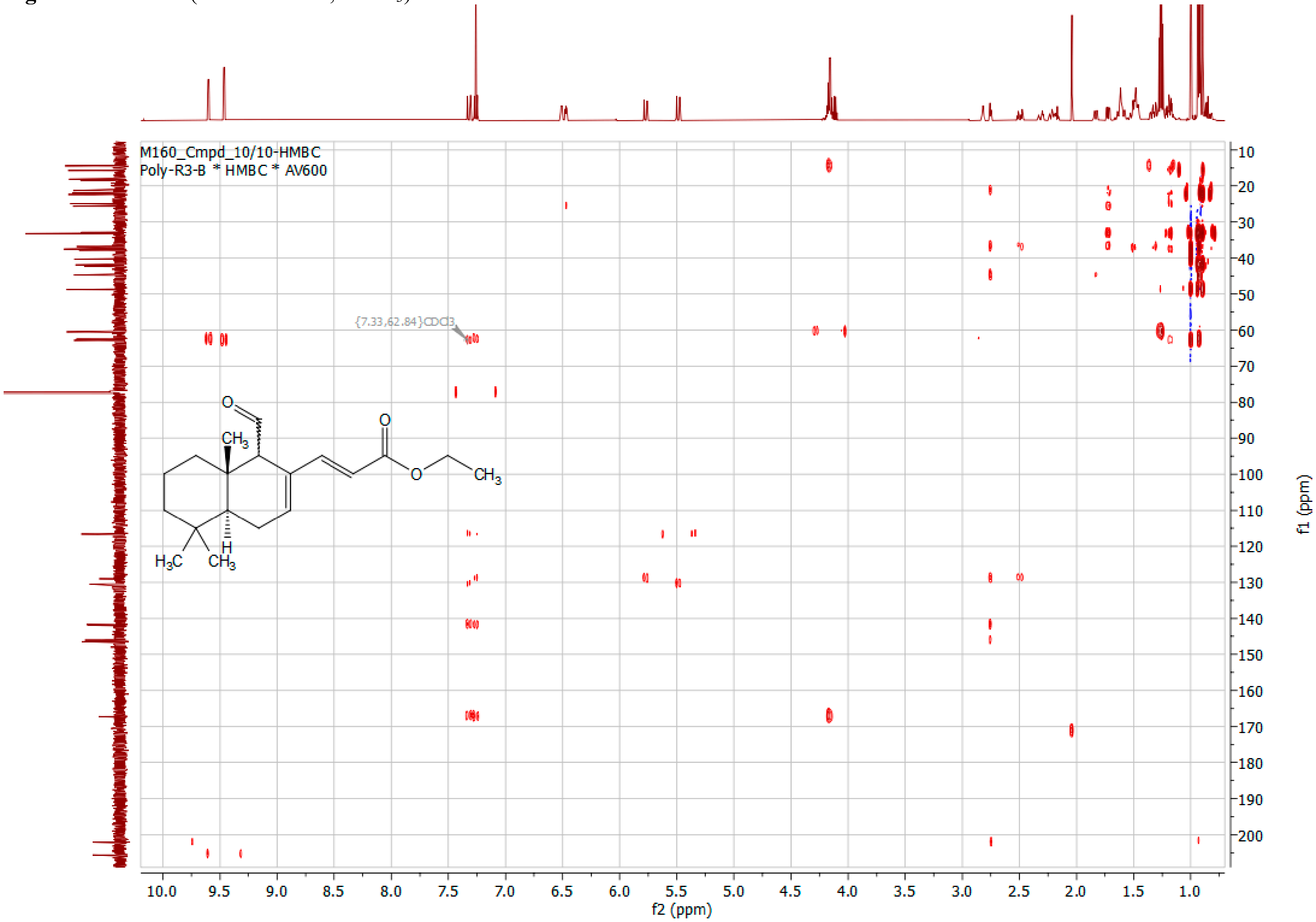

Figure S6: ^{1}H NMR (500 MHz, $CDCl_3$) of **12a**.

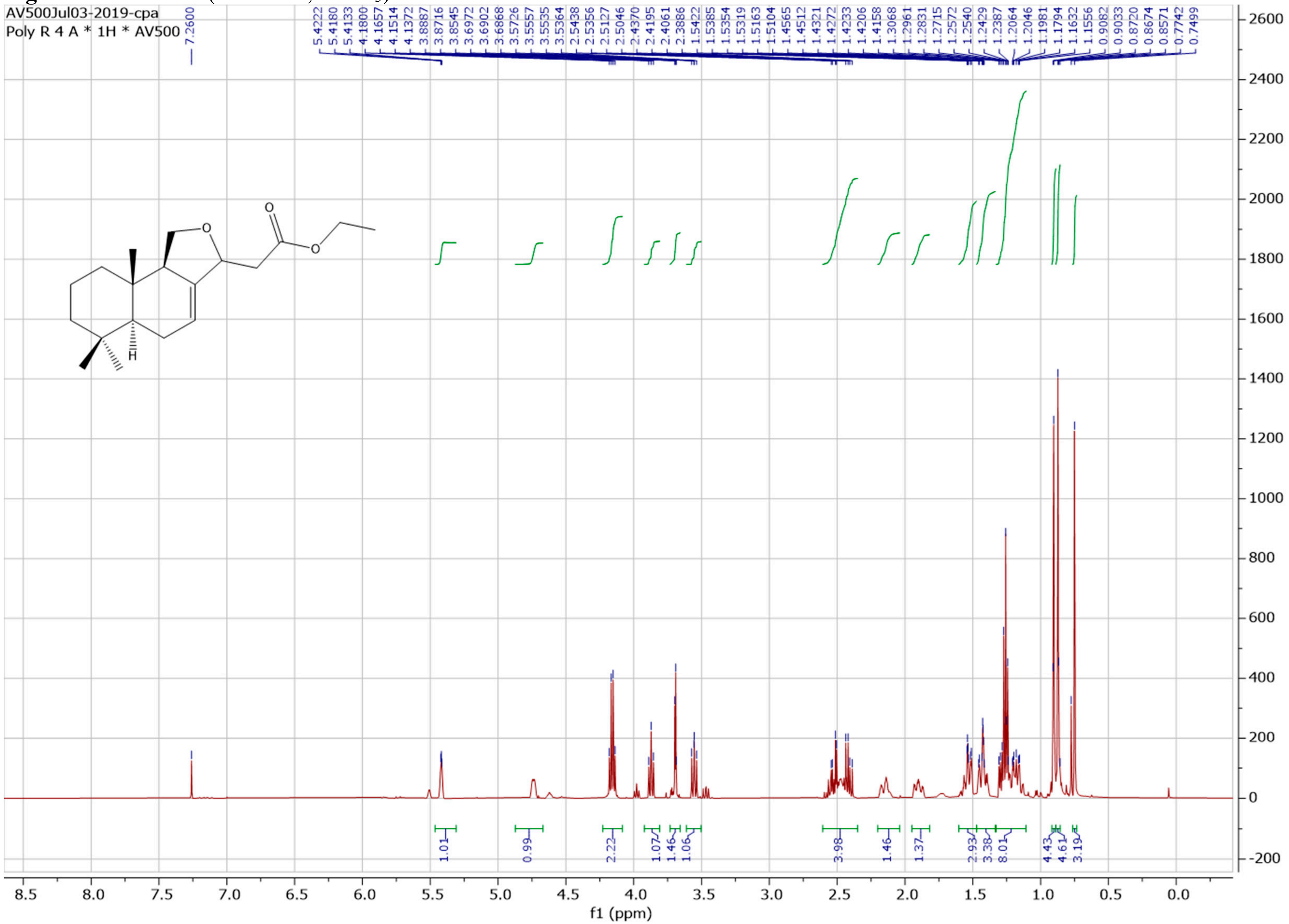

Figure S7: $^{13}C\{^{1}H\}$ NMR (150 MHz, $CDCl_3$) of **12a**.

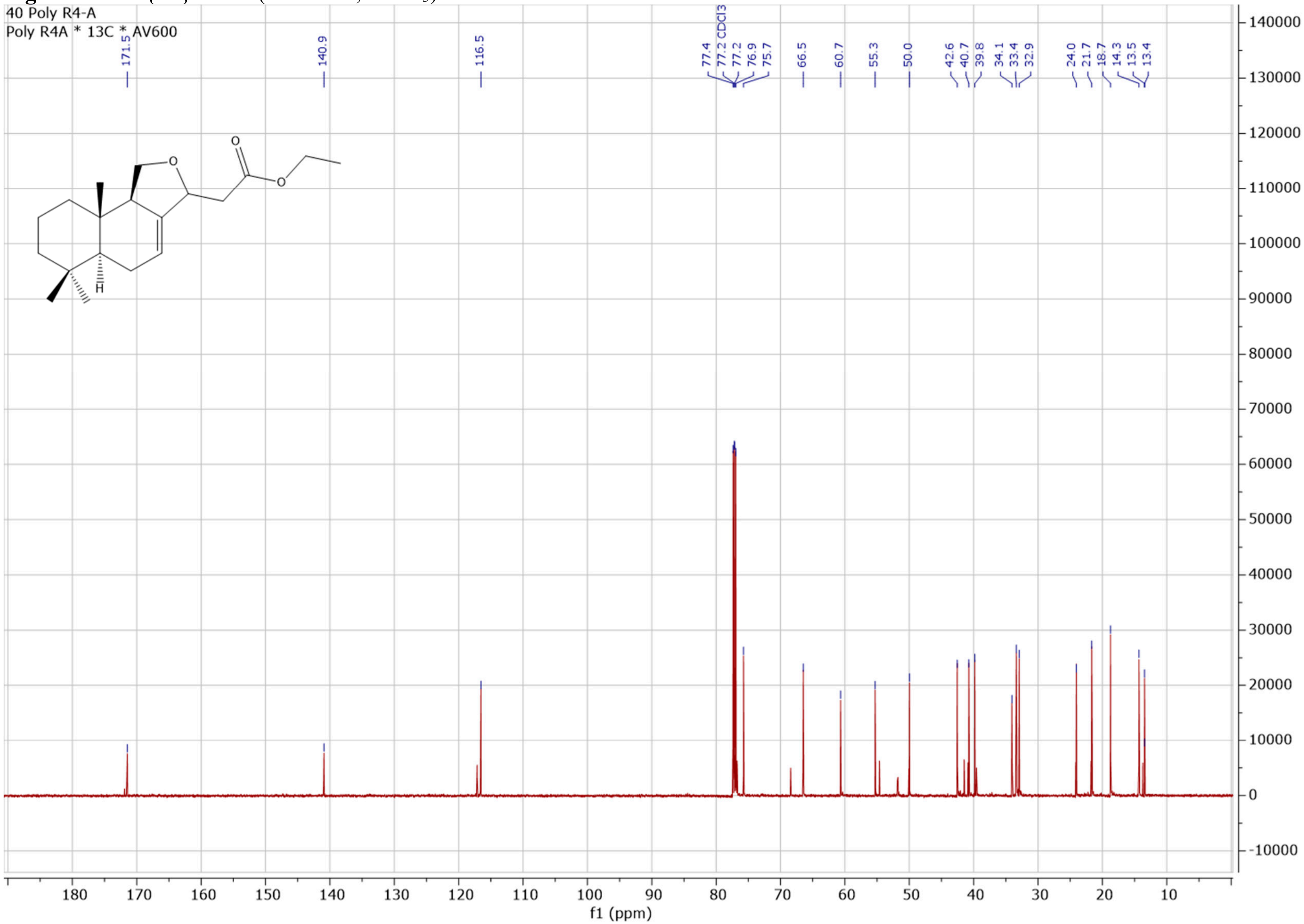

Figure S8: H,H-COSY (500 MHz, $CDCl_3$) of **12a**.

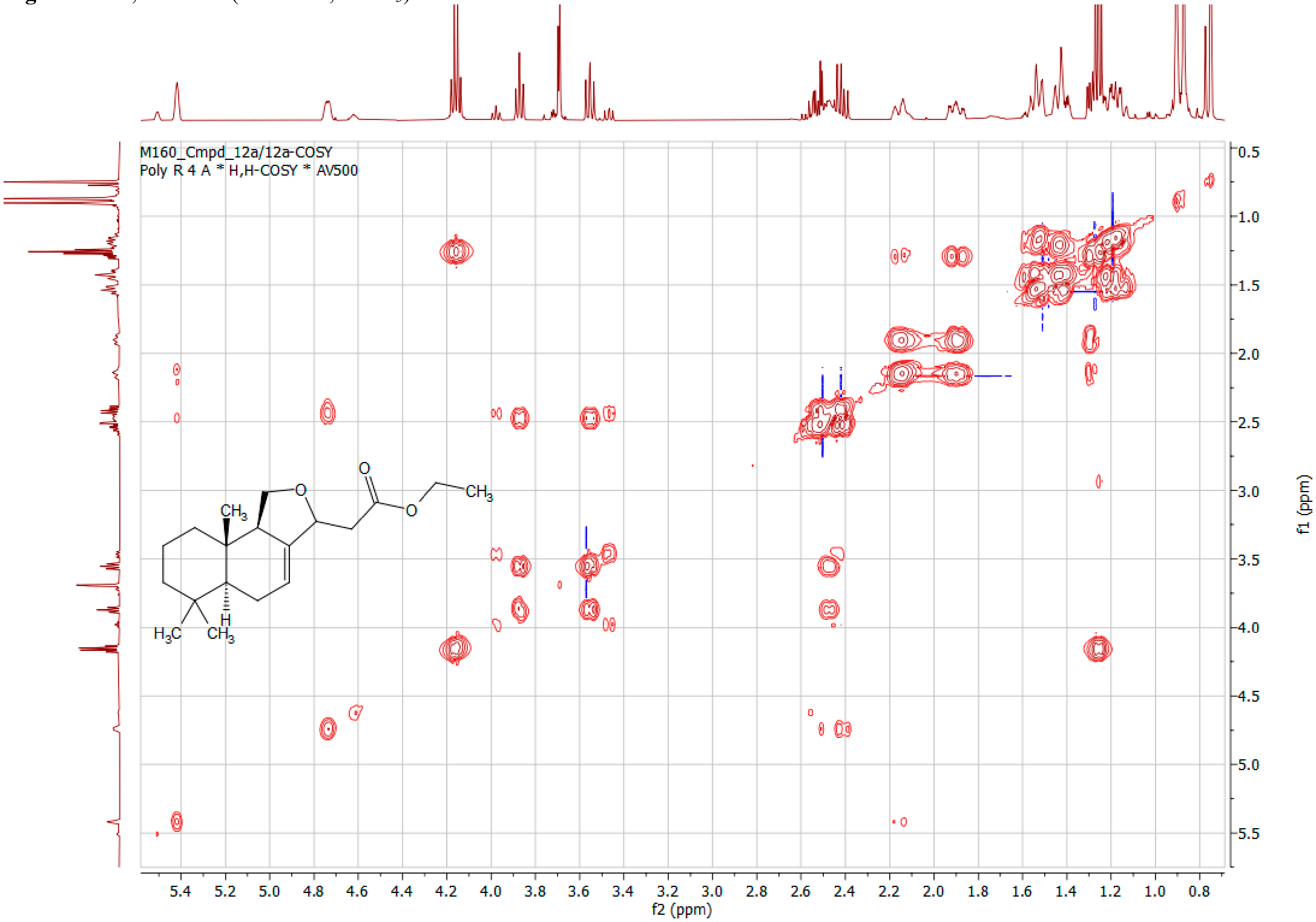

Figure S9: HSQC (500/125 MHz, $CDCl_3$) of **12a**.

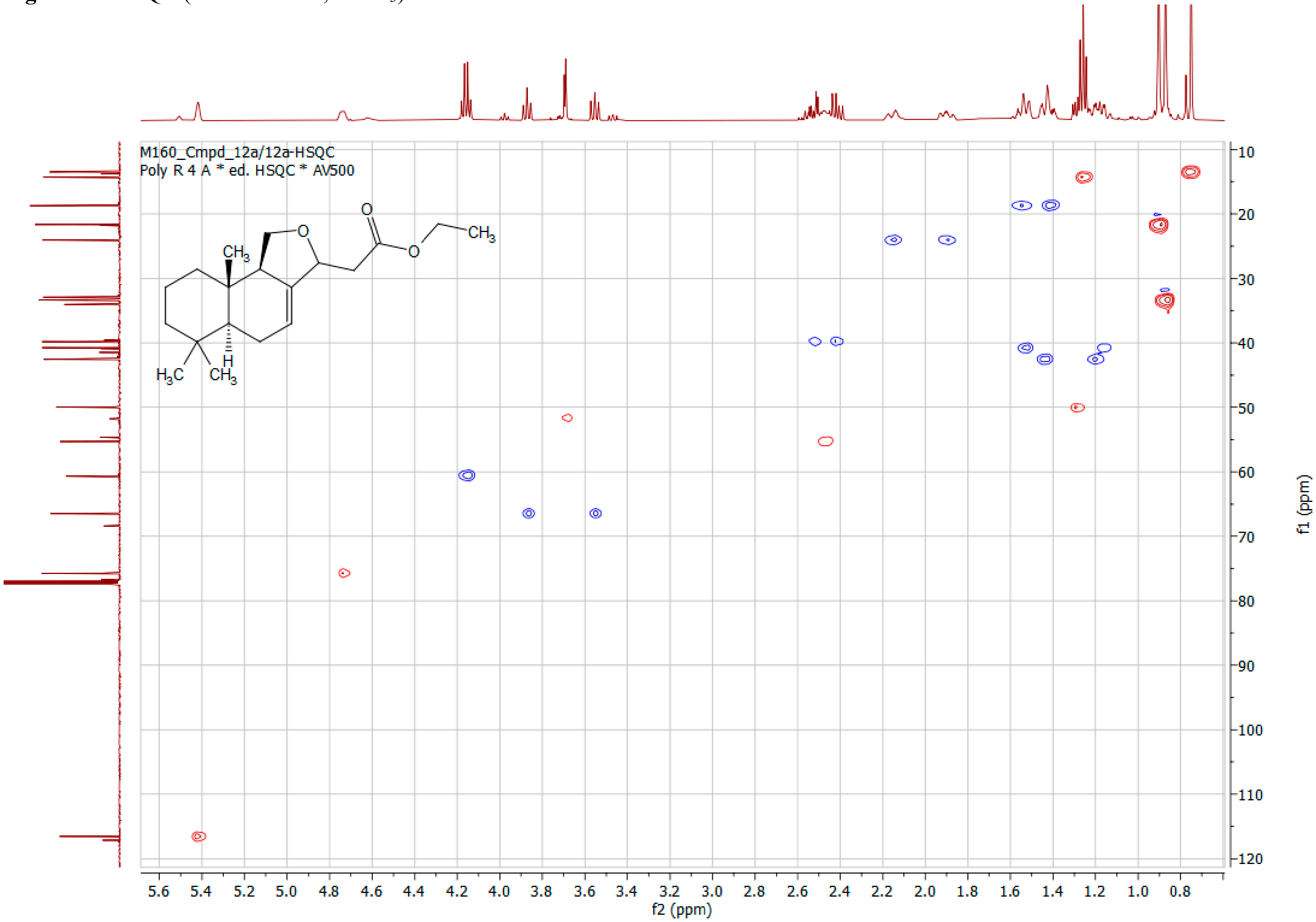

Figure S10: HMBC (500/125 MHz, $CDCl_3$) of **12a**.

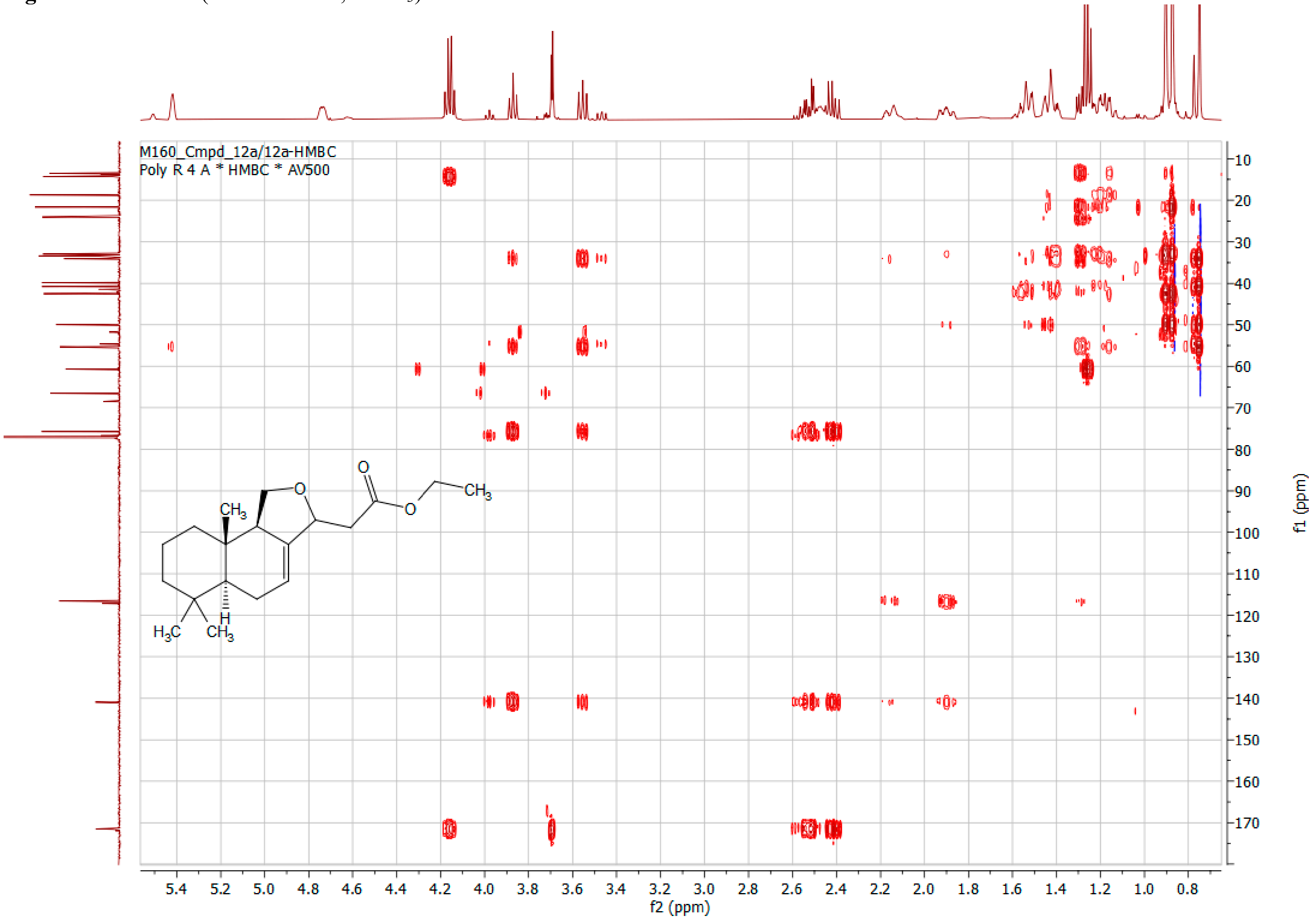

Figure S11: NOESY (500 MHz, $CDCl_3$) of **12a**

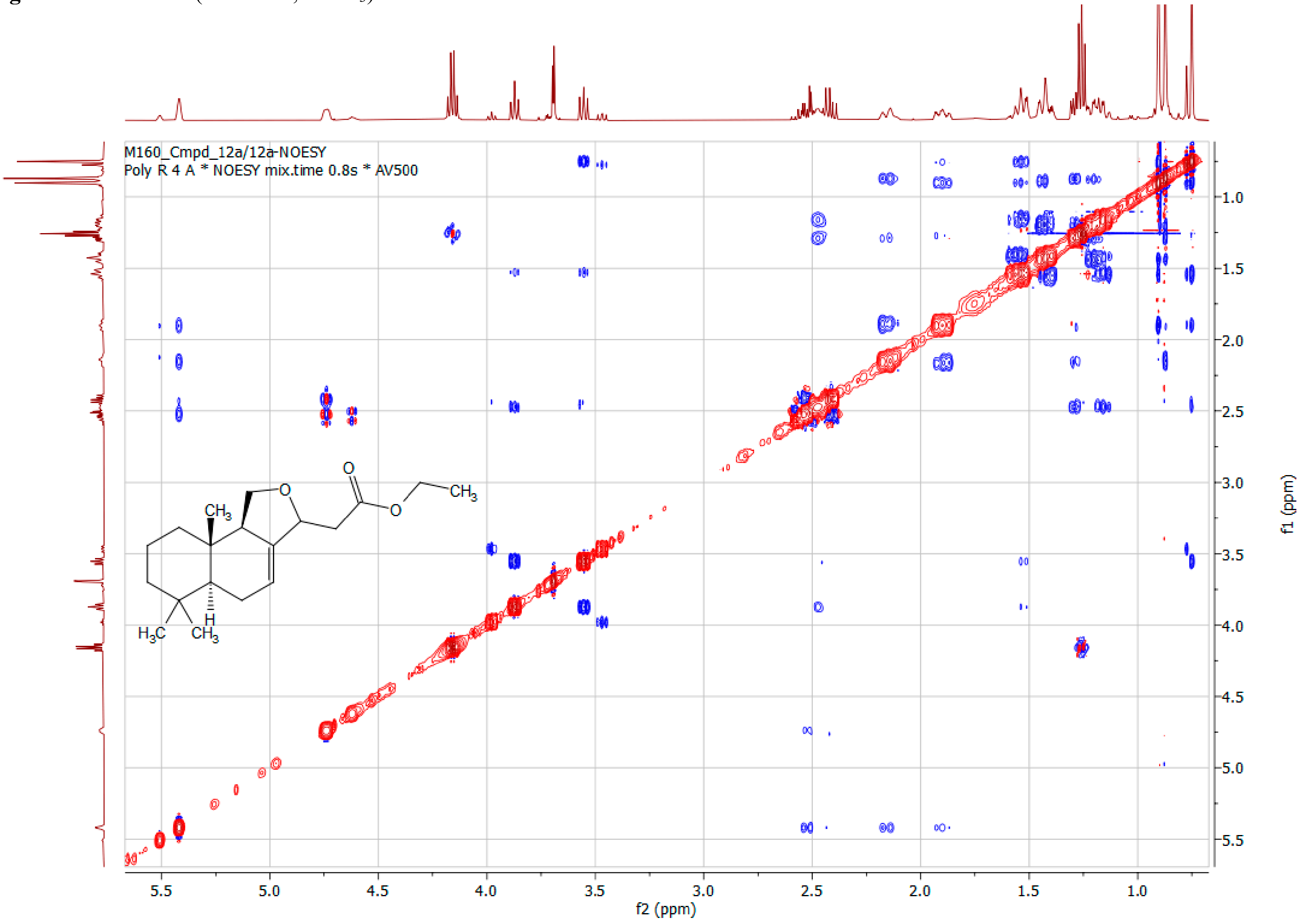

Figure S12: ^{1}H NMR (500 MHz, $CDCl_3$) of **12b**.

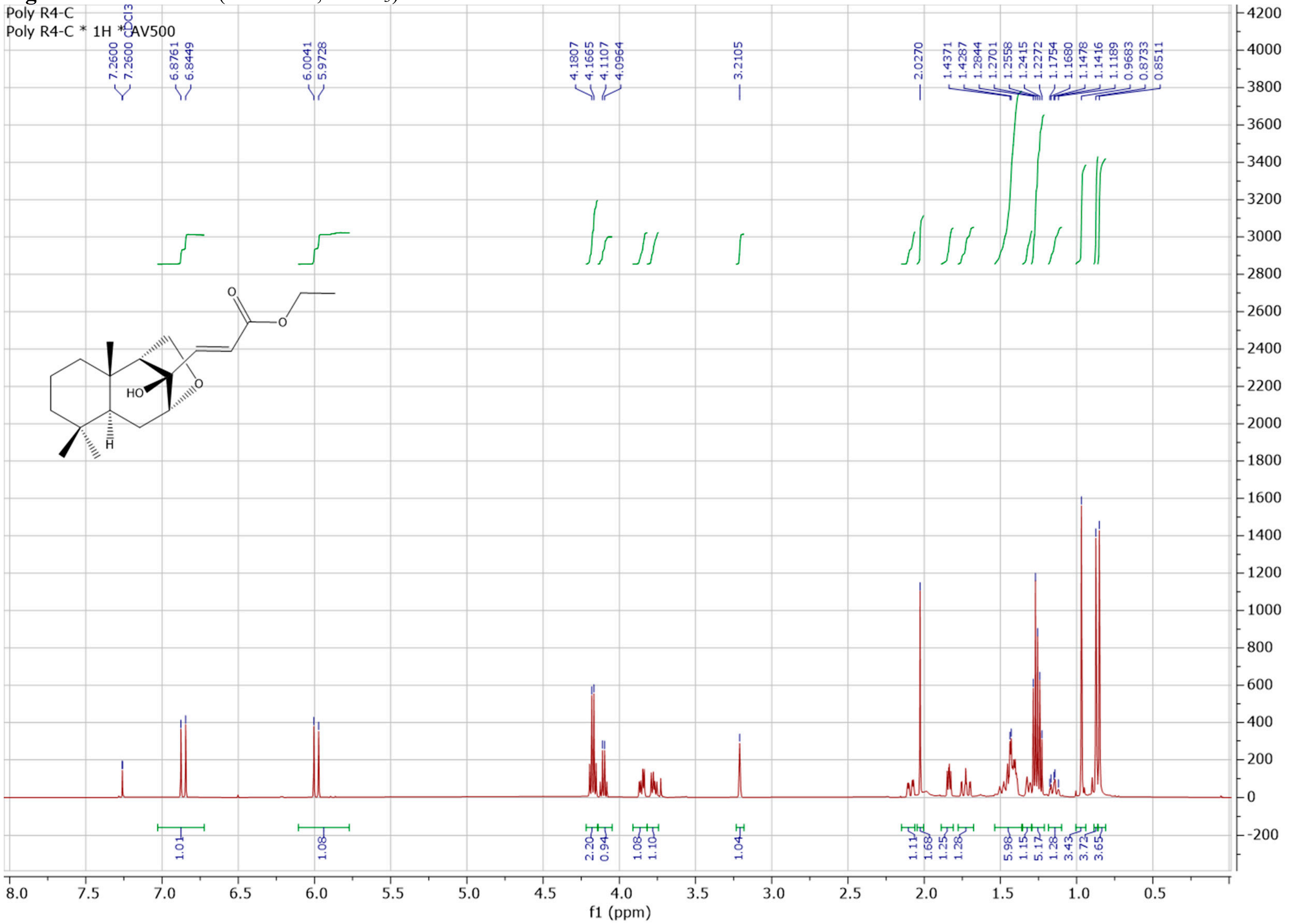

Figure S13: $^{13}C\{^{1}H\}$ NMR (150 MHz, $CDCl_3$) of **12b**.

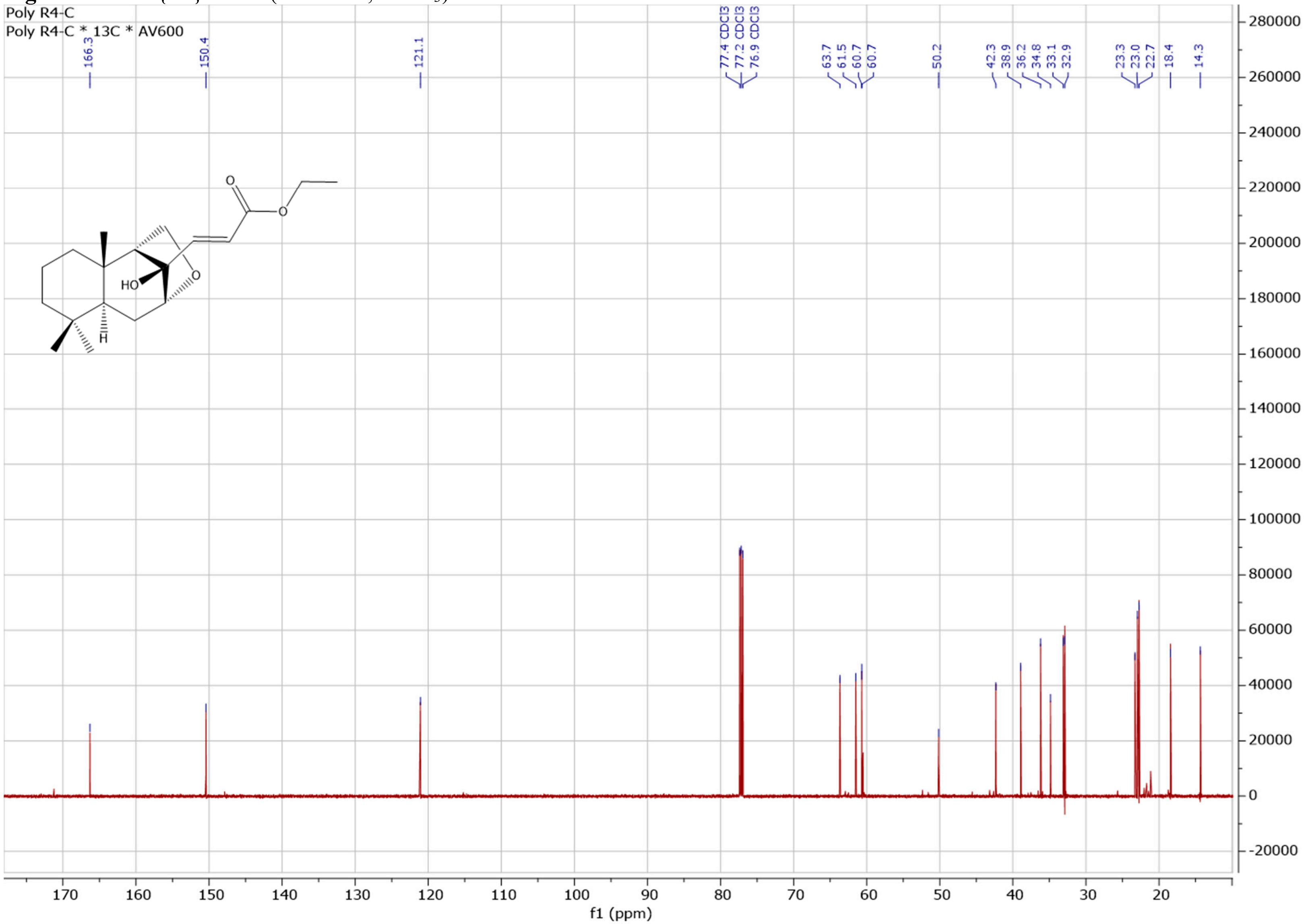

Figure S14: H,H-COSY (500 MHz, $CDCl_3$) of **12b**.

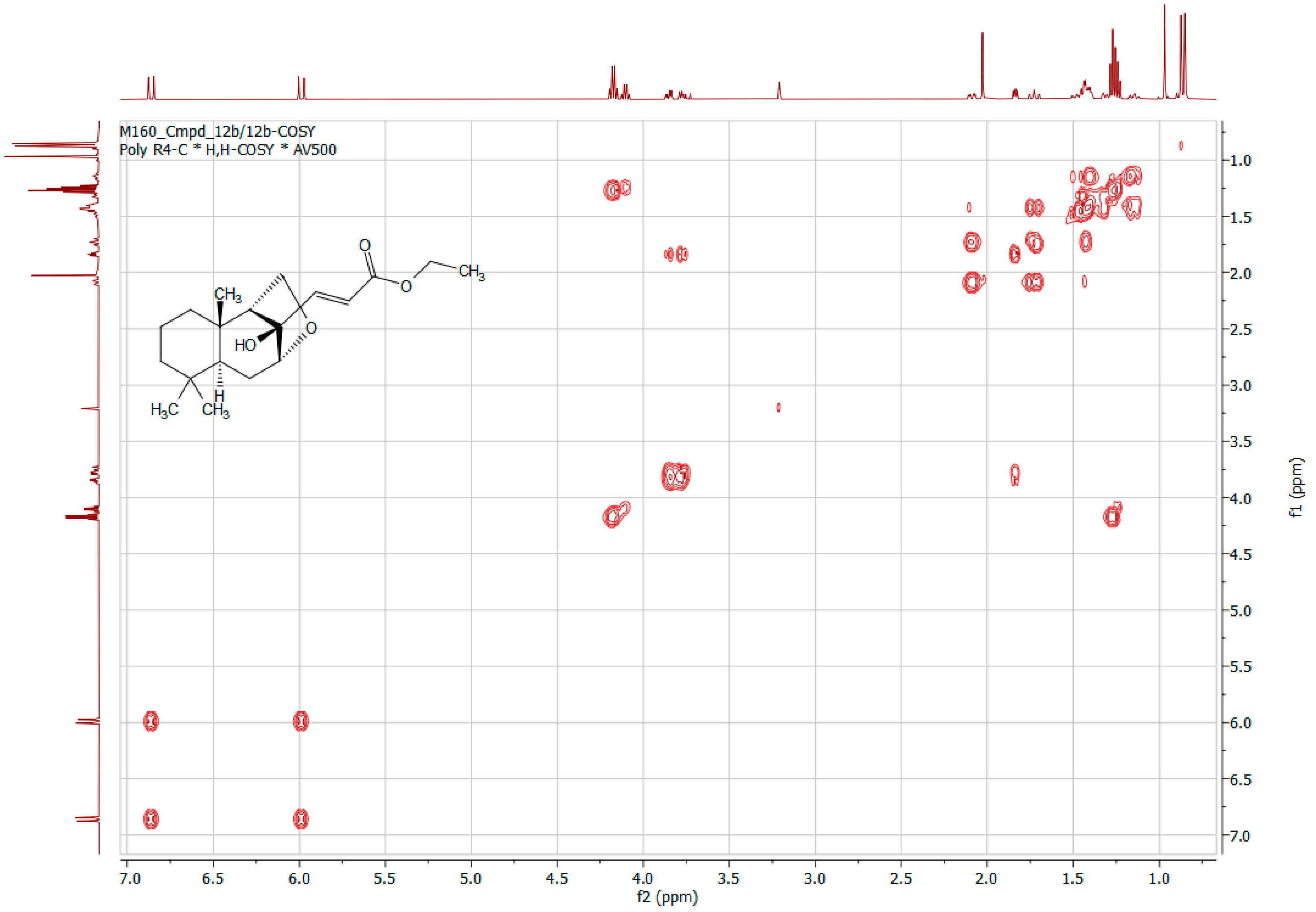

Figure S15: HSQC (500/125 MHz, $CDCl_3$) of **12b**.

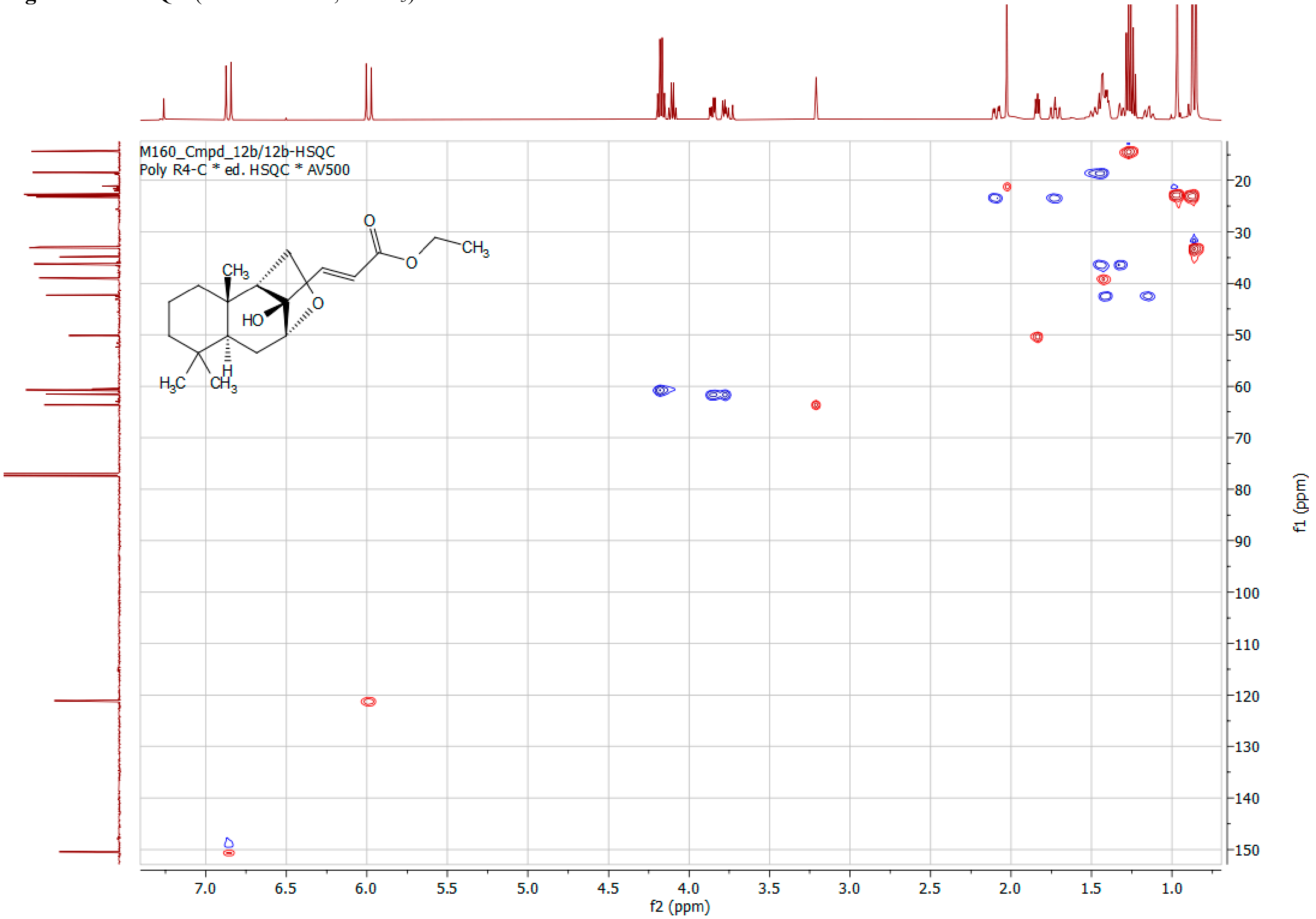

Figure S16: HMBC (500/125 MHz, CDCl$_3$) of **12b**.

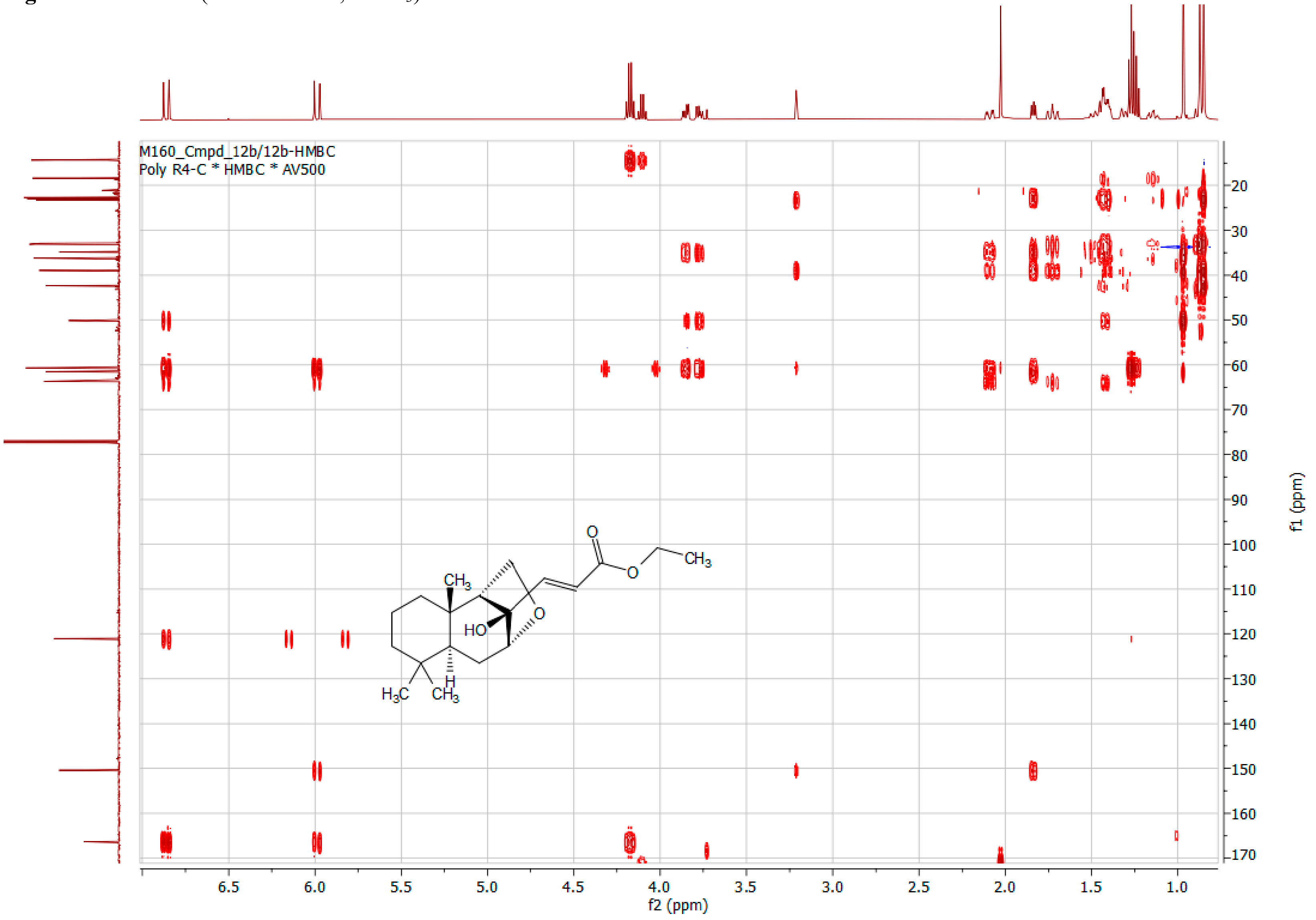

Figure S17: NOESY (500 MHz, $CDCl_3$) of **12b**

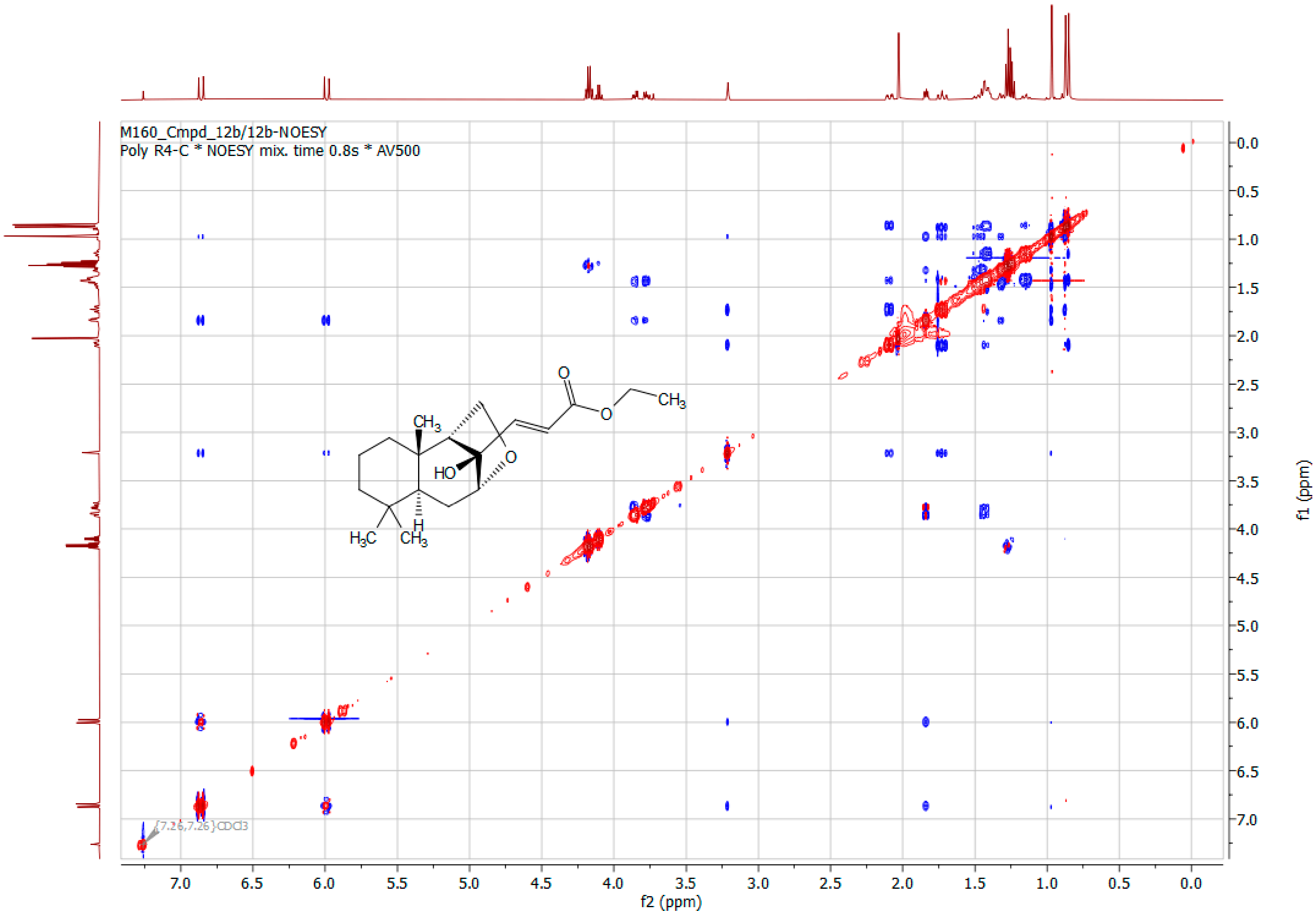

Figure S18. Two-dimensional interaction diagrams and three-dimensional binding poses with α-amylase (PDB 1HNY): (**A**) polygodial (**9**), (**B**) **12a** and (**C**) **12b**.

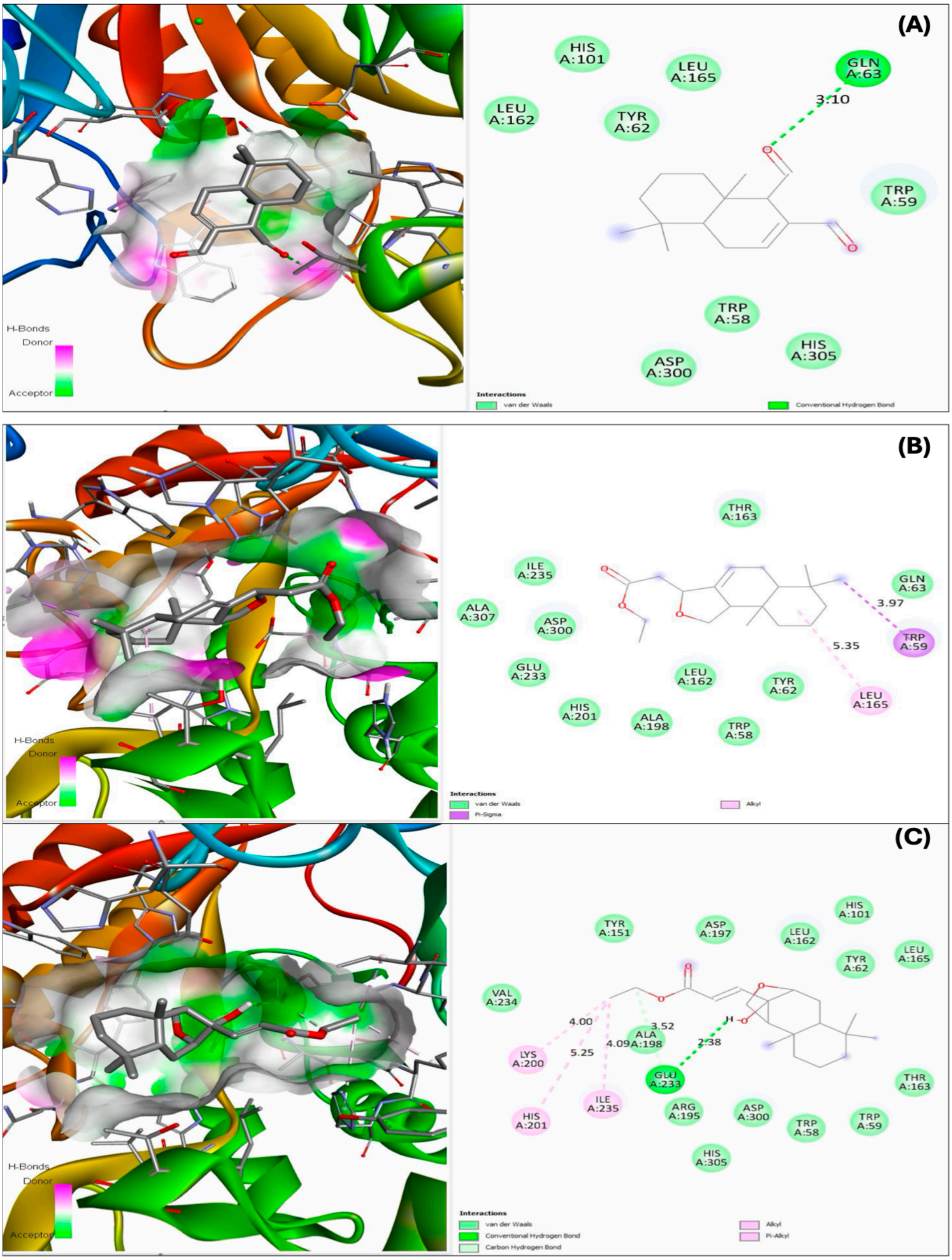

Figure S19. Two-dimensional interaction diagrams and three-dimensional binding poses with α-glucosidase (PDB 3TOP): (A) polygodial **(9)**, (B) **10** and (C) **12b.**

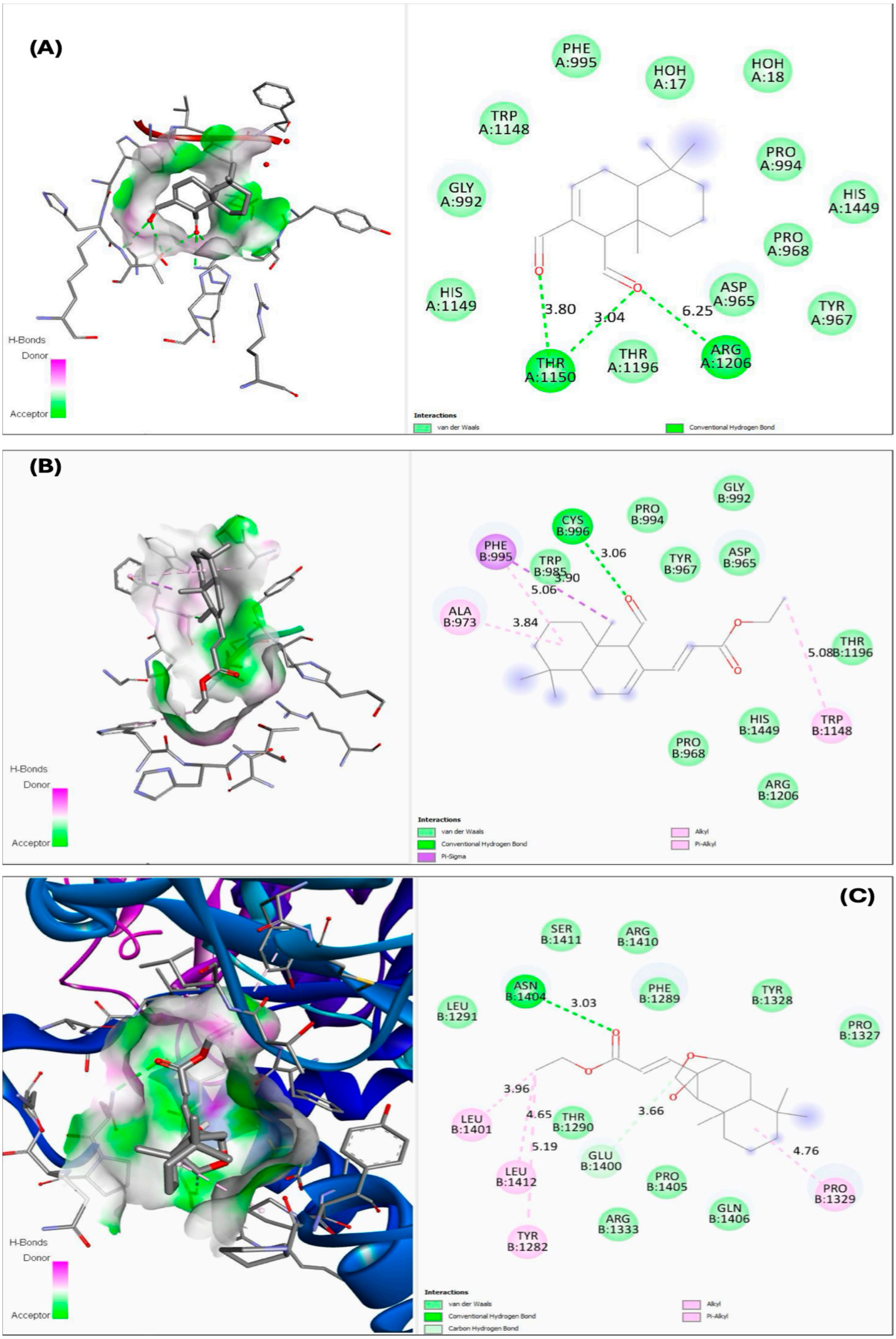

Figure S20. Validation of the docking protocol. The top-ranked pose of acarbose redocked into α-glucosidase (green) superimposed on the crystallographic ligand from 3TOP (grey); heavy-atom RMSD 1.45 Å.

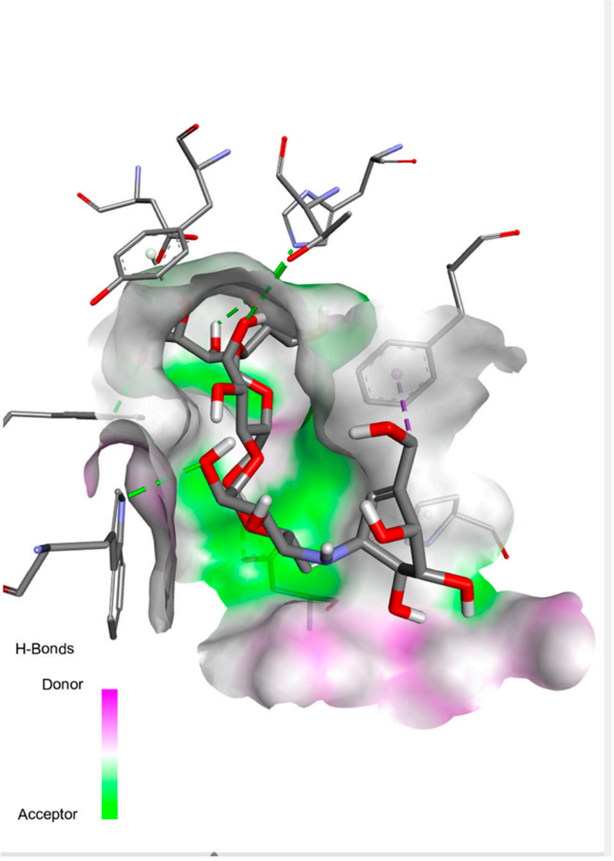

Figure S21. Docked poses of **10**, **12a** and **12b** in α-glucosidase, shown together with the crystallographic acarbose from 3TOP. All structures are in the same reference frame. Only **12a** occupies the acarbose-binding site; the remaining ligands are located at other regions of the receptor surface.

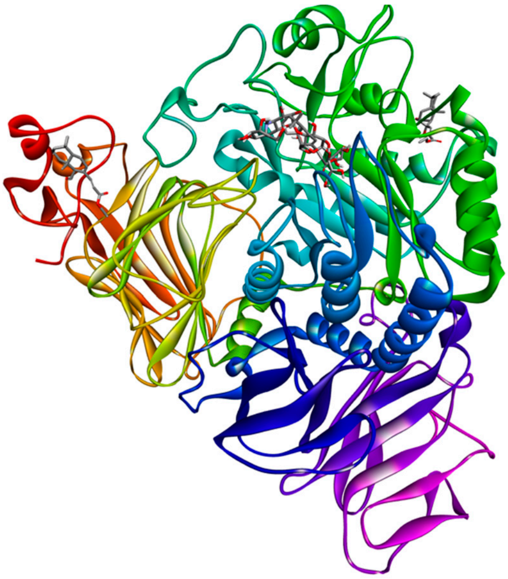

Figure S22. Relationship between measured inhibitory potency and calculated binding affinity for the four drimane compounds: (**A**) α-amylase and (**B**) α-glucosidase. Log_{10} IC_{50} is plotted against docking score; Spearman ϱ and Pearson *r* are given in Table S2.

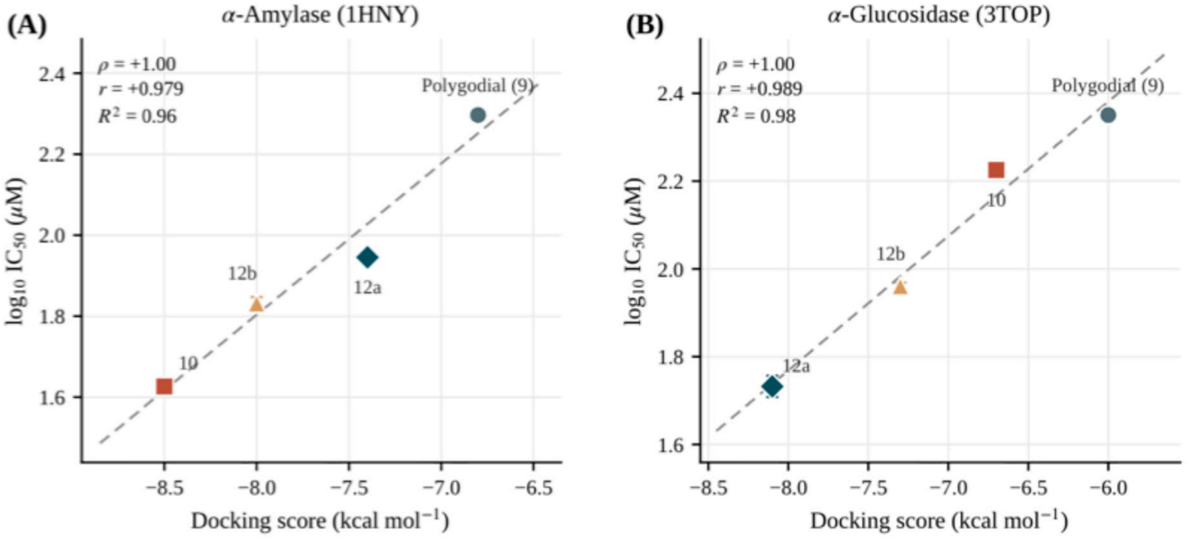

Table S1. Composition of the simulated systems.

System	Total atoms	Water molecules	Na^+	Cl^-	Simulation time (ns)
10 / 1HNY (α-amylase)	20,353	4,208	14	12	200
12a / 1HNY (α-amylase)	20,313	4,194	15	12	200
12a / 3TOP (α-glucosidase)	39,591	8,512	31	24	200

All systems were solvated with the TIP3P water model in orthorhombic boxes, neutralised and adjusted to 0.15 M NaCl.

Table S2. Correlation between measured inhibitory potency and calculated binding affinity for the four drimane compounds.

Enzyme	Spearman ϱ (IC_{50} vs ΔG)	Pearson r (log_{10} IC_{50} vs ΔG)	R^2
α-Amylase (1HNY)	+1.00	+0.979	0.96
α-Glucosidase (3TOP)	+1.00	+0.989	0.98

n = 4 (polygodial, 10, 12a, 12b). With four compounds the exact two-sided probability of a perfect rank agreement arising by chance is 0.083; the coefficients are therefore descriptive rather than inferential. Including acarbose as a fifth point gives ρ = +0.90 for both enzymes, and r = +0.885 (α-amylase) and +0.951 (α-glucosidase).

Table S3. Trajectory metrics for the simulated complexes over 200 ns.

System	Protein Cα RMSD (Å)[a]	Ligand RMSD on protein (Å)[a]	Ligand internal RMSD (Å)[a]	Ligand SASA (Å²)[b]	Binding-site RMSF (Å)
10 / 1HNY	1.90 ± 0.08	9.10 ± 1.98	1.14 ± 0.21	173 → 171	1.08
12a / 1HNY	1.75 ± 0.10	7.46 ± 1.39	1.32 ± 0.38	192 → 168	1.00
12a / 3TOP	2.91 ± 0.09	40.10 ± 14.49	1.45 ± 0.41	219 → 315	—

[a] Mean ± SD over the final 100 ns. [b] Mean over 0–40 ns → mean over 100–200 ns. For the 12a–α-glucosidase system the ligand left the binding site after approximately 110 ns; the values shown reflect that dissociation and this system was excluded from the interaction and collective-motion analyses.

Table S4. Protein–ligand contact occupancies for the two α-amylase complexes, expressed as the percentage of the 200 ns trajectory in which each contact was present.

Complex	Contact type	Residue	Occupancy (%)
10 / 1HNY	Hydrophobic	Trp59	30.3
	Hydrogen bond	Trp58	22.1
	Hydrophobic	Trp58	11.5
	Water bridge	Trp58	14.8
	Water bridge	Asp356	13.2
	Hydrophobic	Leu165	12.1
	Hydrophobic	Tyr151	12.0
	Hydrophobic	Tyr62	9.8
	Hydrophobic	Leu162	9.6
12a / 1HNY	Hydrophobic	Trp59	48.9
	Hydrophobic	Trp58	23.4
	Hydrogen bond	Gln63	18.0
	Water bridge	Thr163	11.3

	Water bridge	Gln63	10.7
	Hydrophobic	Leu165	9.7
	Hydrophobic	Tyr62	9.6
	Water bridge	Asp356	7.2
	Hydrogen bond	Thr163	5.4

Contacts present in more than 5% of frames are listed. Occupancies were extracted with the Schrödinger Simulation Interaction Diagram tools.

Table S5. Variance captured by the leading principal components of the Cα coordinates for the two α-amylase complexes.

**Complex**	**PC1 (%)**	**PC2 (%)**	**PC3 (%)**	**PC1–PC3 cumulative (%)**
10 / 1HNY	28.7	9.3	5.0	42.9
12a / 1HNY	16.9	13.1	6.0	36.0

Approximately seven components are required to account for half the total fluctuation in each system. The 12a–α-glucosidase trajectory was excluded, since the ligand left the binding site during the simulation.
